# Metalloprotein-Specific or Critical Amino Acid Residues: Perspectives on Plant-Precise Detoxification and Recognition Mechanisms under Cadmium Stress

**DOI:** 10.3390/ijms23031734

**Published:** 2022-02-03

**Authors:** Dandan Li, Tengbing He, Muhammad Saleem, Guandi He

**Affiliations:** 1 College of Agricultural, Guizhou University, Guiyang 550025, China; gs.lidd19@gzu.edu.cn (D.L.); tbhe@gzu.edu.cn (T.H.); 2 Institute of New Rural Development, West Campus, Guizhou University, Guiyang 550025, China; 3 Department of Biological Sciences, Alabama State University, Montgomery, AL 36104, USA; msaleem@alasu.edu

**Keywords:** Cd transport and detoxification, H/DXXXD, cysteine-related residues, functional protein

## Abstract

Cadmium (Cd) pollution in cultivated land is caused by irresistible geological factors and human activities; intense diffusion and migration have seriously affected the safety of food crops. Plants have evolved mechanisms to control excessive influx of Cd in the environment, such as directional transport, chelation and detoxification. This is done by some specific metalloproteins, whose key amino acid motifs have been investigated by scientists one by one. The application of powerful cell biology, crystal structure science, and molecular probe targeted labeling technology has identified a series of protein families involved in the influx, transport and detoxification of the heavy metal Cd. This review summarizes them as influx proteins (NRAMP, ZIP), chelating proteins (MT, PDF), vacuolar proteins (CAX, ABCC, MTP), long-distance transport proteins (OPT, HMA) and efflux proteins (PCR, ABCG). We selected representative proteins from each family, and compared their amino acid sequence, motif structure, subcellular location, tissue specific distribution and other characteristics of differences and common points, so as to summarize the key residues of the Cd binding target. Then, we explain its special mechanism of action from the molecular structure. In conclusion, this review is expected to provide a reference for the exploration of key amino acid targets of Cd, and lay a foundation for the intelligent design and breeding of crops with high/low Cd accumulation.

## 1. Introduction

Cd is a naturally occurring environmental toxicant, which is easily absorbed and accumulated by plants, and has strong teratogenic and mutagenic effects on organisms. Human exposure to Cd for a long time can easily cause diseases such as osteoporosis [1] and kidney damage [2]. It is also positively correlated with the outbreak of a variety of cancers [3], and is classified as a human carcinogen by the International Agency for Research on Cancer (Group I) [4]. A large amount of data shows that the intake of Cd in the human body is mainly from the diet [5]. Therefore, it is very important to understand the influx and transport mechanism of Cd in plants.

The Cd enters the soil through man-made activities such as Zn mining, smelting, application of fertilizers and pesticides [6]. When the content of Cd in the soil is too high, it will inhibit the influx of iron by plants, causing plant growth retardation, chlorosis and other symptoms similar to iron deficiency [7,8]. These symptoms seem to be caused by the direct or indirect interaction between Cd and Fe. Cd toxicity may also cause phosphorus deficiency or reduce manganese input, and interfere with the influx and transport of several essential nutrients (such as calcium, magnesium, phosphorus and potassium) and water by plants [9]. In summary, Cd can enter the cell through essential element transporters. Due to the lack of specificity of these transporters, Cd, as a non-essential element, is usually assumed to be absorbed by transporters of essential elements (such as Zn, Fe, and Ca) [10,11]. Most of the early literature also pointed out that the transporter-mediated symbiotic and the coupled transcellular pathway is the only way for Cd to flow in [12,13]. The members of the natural resistance-associated macrophage protein (NRAMP), ZRT, IRT-like protein (ZIP), metallothionein (MT) and plant defensins (PDF), cation/proton exchanger (CAX), ATP-binding cassette transporters (ABC), metal tolerance protein/cation diffusion facilitator (MTP/CDF), heavy metal ATPase (HMA), oligopeptide transporter (OPT) and plant Cd resistance (PCR) families are closely related to the influx, chelation, transport and efflux of Cd in plants.

**Figure 1:**
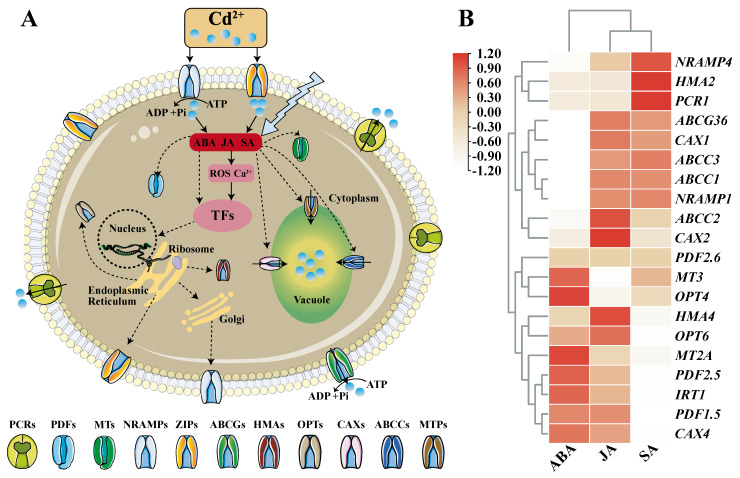
Plant hormone signaling regulation and related gene expression analysis under Cd stress. A: The regulatory pathway of plant hormones under Cd stress. Plant hormones such as ABA, JA, SA enhance the detoxification ability of plants against cadmium stress by regulating the expression of downstream proteins. TFs stands for transcription factors. The arrows with solid lines indicate that the regulation methods have been reported so far, and the arrows with dotted lines indicate that the regulation methods are not yet clear. Polyline arrows indicate exogenous additions. B: Effects of ABA, JA and SA on the expression of genes involved in Cd transport and detoxification (taking Arabidopsis as an example). Expression data comes from the database (http://ipf.sustech.edu.cn/pub/athrna/) (accessed on 30 December 2021). A red box means more correlation, a white box means less correlation. Plant hormones like ABA, JA, SA are closely related to the expression of these genes.

When plants are stressed by Cd, plant hormones such as abscisic acid (ABA), jasmonic acid (JA), and salicylic acid (SA) will increase and have an anti-stress effect. It was reported that exogenous application of growth regulators like ABA, JA and SA, chemical regulators and nutrient management (application of phosphorus, silicon, etc.) can improve plant tolerance to cadmium [14]. By integrating the data in http://ipf.sustech.edu.cn/pub/athrna/ (accessed on 30 December 2021), it was found that ABA, JA, SA hormones are closely related to the expression of cadmium transport and detoxification genes (Figure 1). Studies on their mechanism of action have found that they can act as signaling factors to up-regulate the expression of cadmium-tolerant protein genes or down-regulate the synthesis of absorption and transport proteins, thereby improving the detoxification ability of plants to cadmium (Figure 1) [15]. For example, ABA can inhibit the expression of Cd uptake genes and lower Cd accumulation [16], and SA significantly reduces Cd in rice grains by regulating the expression levels of genes associated with Cd translocation and accumulation, such as *OsNRAMP2* and *OsHMA3* [17]. JA inhibits the expression of genes that promote Cd uptake and long-distance translocation, positively limits Cd accumulation and alleviates Cd toxicity in Arabidopsis through the jasmine acid signaling pathway [18]. Similarly, the significant effect of Si in reducing Cd accumulation has also been attributed to its down-regulation of the expression of genes involved in Cd uptake (*OsNramp5*) and root-to-shoot translocation (*OsHMA2*) [19]. Exogenous addition of astaxanthin and its gold nanoparticles [20], β-cyclocitral [21], glutamic acid [22], nitric oxide [23], etc. increased the tolerance of plants to Cd stress, which is also because they alter the expression of MTP, HMA, OPT, ZIP, NRAMP, ABC and other family members. In the main, the influx, transport and detoxification mechanism of Cd by plants are closely related to proteins.

As so often is the case, the realization of biological functions depends on the interaction between ligand binding residues and metal ions, and the molecular mechanism involves the binding of metal ions to specific residues in the protein. Some of these amino acid motifs are conserved in the protein family, and they can produce key stable interactions or play important functional roles [24]. Each family has its own conserved motifs, which play an important role in protein recognition and metal ion binding.

In this review, in order to understand the main functions of these family proteins in rice and Arabidopsis, they are divided into influx proteins, chelating proteins, vacuolar proteins, long-distance transport proteins and efflux proteins. Two or more functional proteins in each family are discussed, comparing their mechanism of action and protein sequence similarities and differences. The Cd uptake and translocation of plant roots to the ground is analyzed, and how these proteins can increase plant tolerance to Cd toxicity or reduce the mode of action of Cd accumulation is summarized. Determining the key residues that interact directly or indirectly with Cd provides a new idea for reducing the Cd content in plants and the safe production of food crops.

## 2. Influx Protein

There are two different processes in the influx of Cd by plant roots. The first is a temperature-independent process, which may be passive, reaching an equilibrium concentration after a few seconds or minutes. The second is the physiological process, which mainly depends on the relevant transport proteins in plants to play a role [25]. There is no Cd selective transporter in plant cells. Cd in soil enters plant cells mainly through plasma membrane transporters such as NRAMP and ZIP [26].

### 2.1. A Brief Introduction to the NRAMP Family, the Key Proteins Related to Cd Influx and the Mechanism of Action

NRAMP, a proton-coupled metal ion transporter, is an ancient intact membrane transporter family. It is widely present in bacteria, fungi, plants and animals, and participates in the transportation of a variety of divalent metal ions [27]. Using online software (http://wlab.ethz.ch/protter/start/) (accessed on 30 December 2021) to map their transmembrane domains (TMDs) and amino acid structural features [28], it was found that they typically have 12 TMDs. The DPGN residue near TMD1 was identified as the binding site of the NRAMP transporter (Figure S1), which coordinates with divalent metal ions and participates in the transport of Cd to plant root cells [29]. Mutations in these residues can cause impaired transporter function. In Arabidopsis, a total of 6 NRAMP members have been identified, of which AtNRAMP1, AtNRAMP3 and AtNRAMP4 are related to the influx and transport of Cd [30]. Yeasts and Arabidopsis that overexpress these genes can increase their sensitivity to Cd and contain more Cd in yeast cells. There were seven NRAMP genes found in rice. Among these members, OsNramp1 and OsNramp5 were identified as the main Cd uptake protein in rice [31]. It is interesting that even in the same species, NRAMP has undergone some interesting changes to regulate the accumulation of heavy metals. For example, the amino acid sequence of OsNRAMP1 in high and low accumulation rice varieties is exactly the same, but the Cd content can be reduced by regulating the low expression of OsNRAMP1 in the roots [32]. The transport function of NRAMP family members is driven by H co-transport, H provides energy for active transport, and the conformational changes of proteins are crucial [33]. Residue phenylalanine413 in AtNRAMP4 is located in the highly conserved protein domain of plant and animal NRAMP members, and its mutation can change the accumulation of Cd in plants [34]. The mutations of histidine in AtNRAMP3 severely impaired the transport of Fe and Mn, and partially impaired the transport of Cd [35].

### 2.2. A Brief Introduction to the ZIP Family, the Key Proteins Related to Cd Influx and the Mechanism of Action

The ZIP family mainly comes from dicotyledonous plants, which play a major role in the homeostasis of zinc and metal in plants [36]. They are not highly selective and can transport different metal divalent cations, including essential elements (such as Zn, Mn, Fe) and toxic elements (Cd) [37]. The iron-regulated transporter 1 (IRT1) is the main root transporter that absorbs iron from the soil, and is also the main entry route for potentially toxic metals such as manganese, zinc, cobalt, and Cd in plants [38,39]. For example, AtIRT1 targets the plasma membrane in the body, which can mediate the influx of Cd from the environment [40]. When AtIRT1, OsZIP1 and OsZIP3 are expressed in yeast cells, these three genes increase the sensitivity of yeast to Cd stress and the accumulation of Cd [41]. In rice, overexpression of OsIRT1 and OsIRT2 made cells more sensitive to Cd and increased Cd accumulation [42]. OsZIP6 can transport at least three transition metal ions such as Fe, Co and Cd [43]. Studies have found that most ZIP proteins have 8 potential TMDs and a similar membrane topology (Figure S2), and the amino and carboxyl terminal ends of the protein are located on the outer surface of the plasma membrane [44]. The length of the ZIP protein varies from 309 to 476 amino acids [45]. The most conserved part of the ZIP family proteins appears in TMD IV, which is expected to form an amphipathic helix with completely conserved histidine residues. The histidine residue, together with the adjacent (semi) polar residues, can form part of the heavy metal binding site in the membrane as a transport pathway [46]. Understanding the mechanism of ZIP transporters requires their high-resolution crystal structure, which is not yet available for plant ZIP protein. However, it was found in *Bordetella bronchiseptica* that the crystal structure of the BbZIP transporter has 8 TMDs and a binuclear metal center (M1, M2) was observed [47]. M1 may be located just on the transportation route, which can quickly combine and release metals during transportation, which is necessary for the transportation of metal ions, and M2 may play a supporting role [48]. Compared with BbZIP, plant ZIP transporters may have partially overlapping but different metal transport mechanisms, because some residues of ZIP involved in metal binding and transport are not conserved in plants [49].

### 2.3. Comparison of Core Characteristics of Cd Influx Key Proteins

Compared to the key proteins for Cd uptake in rice and Arabidopsis, it was found that these proteins were mainly expressed in plant roots and located on the cell membrane, indicating that the free Cd in the soil solution was mainly transported into the plant through root transporters (Table 1). The NRAMP protein mainly relies on H co-transport drive to provide energy, while the transport mechanism of ZIP is currently unclear. In particular, there are only about 5 NRAMP members whose evolution tends to be conservative in plants, and there is no large-scale expansion. DPGN residues are only found in NRAMP, which may be a key and special function residue. In animals, members of the NRAMP family have been shown to play an important role in the immune response, usually in the phagocytosis and presentation of antigens by pathogens, but whether there are similar functions in plants has not been reported. In addition to the Cd stress response, it seems likely that it participates in a series of foreign invasion defenses. However, there are many members in the ZIP family and their evolution has undergone specific changes. For example, all of the ZIP proteins involved in Cd influx have a signal peptide at the N-terminus. Signal peptides can play a role in controlling protein secretion rate, determining protein folding state, affecting downstream transmembrane behavior and N-terminal glycosylation, and nuclear localization signals [50]. The changes may lead to differences in subcellular functions between them, and may produce specific stress responses to different metal ions. Between TMD3 and TMD4 of the ZIP protein, there is a histidine-rich residue (HX)n (X = any amino acid, n = 3–6) that can act as a Zn buffer bag and a cytoplasmic surface zinc level sensor, and it may be a potential metal (Zn, Co and/or Cd) binding domain (Figure S2). This residue is also ubiquitous in the MTP family, but surprisingly, the MTP principally produces an effect in the vacuole [51]. Interestingly, OsZIP1 can also be located in the endoplasmic reticulum, while OsZIP3 can be expressed in xylem parenchyma cells. The parenchyma of the xylem of plant roots is the main “controller” of Cd loading into the xylem and its transport to the branches [52], indicating that the functions of the family members have diversified. However, these changes may benefit from the emergence of the N-terminal specific signal peptide of the ZIP family. Future research should aim to determine whether it will play a more unique role, for example, the special signal peptide may be improved to become a part of the NRAMP family.

## 3. Chelating Protein

Only a small part of the metal content in plants exists in the form of free water ions, and most of the ions are combined with low molecular weight ligands or proteins. The cell wall is the first protective barrier to prevent heavy metals from invading cells, the first “living” structure, and the target of heavy metal damage. The immobilization of Cd in the cell wall participates in the tolerance mechanism of plants to Cd, and PDF exists in the cell wall and can participate in the chelation of it. However, MT mainly exists in the cytoplasm and can combine with free Cd to reduce the harm of heavy metals to plants.

### 3.1. A Brief Introduction to the MT Family, Key Proteins and Chelation Mechanism

MT is a low-molecular-weight metalloprotein in cells that is rich in cysteine and exists in all organisms except archaea [58]. According to the distribution pattern of cysteine, it can be divided into 4 sub-families: MT1, MT2, MT3 and MT4 [59]. The MT1-3 subfamily has two cysteine-rich regions, 6 cysteines in the C-terminal domain, and 6, 8 and 4 cysteines in the N-terminal cysteine-rich region, respectively. M4 has three cysteine-rich regions, each containing 6, 6 and 5 cysteine residues [60]. It can be combined with heavy metal Cd with high affinity, and plays a role in detoxification of heavy metals and maintaining the stable state of essential metal ions in cells. Among them, MT1, because of its unique metal bonding characteristics, can endow Cd resistance and contribute to the stable state of zinc [61]. The heterologous expression of OsMTI-1b can improve the tolerance of *E. coli* cells to Cd by chelating metal ions [62]. Overexpression of OsMT1e improved rice growth under Cd stress [63]. After CdCl_2_ treatment, Arabidopsis lines overexpressing OsMT-3a accumulated higher levels of Cd in shoots and roots [64]. From Figure S3, we can see that the primary structure of MT is obviously rich in highly conserved cysteine residues such as CC, CXC and CXXC (X = any amino acid). These residues have the remarkable ability to bind a large number of monovalent or divalent metal ions, as well as maintain the steady-state of basic metals and the ability to detoxify (toxic) metals, especially copper, zinc and Cd [65]. The sulfhydryl groups on MTs can combine with heavy metal ions to form non-toxic or low-toxic compounds, thereby eliminating the toxicity of heavy metals [66]. Furthermore, the mercaptans (esters) in MTs can act as powerful antioxidants. They usually bind metal ions in metal thiol clusters to provide high thermodynamic stability and kinetic instability [67]. MT constitutes a highly complex system, even though the same peptide can show different final 3D folds, it is expected that these folds are related to different functions [68].

### 3.2. A Brief Introduction to the PDF Family, Key Proteins and Chelation Mechanism

PDF is a type of small molecule protein, each containing 45 to 54 amino acids (Figure S4), and 13 members have been identified in Arabidopsis [69]. PDF can be divided into two families, namely PDF1 and PDF2, which mainly play a role in fungal resistance and zinc tolerance, but it is interesting that the recent reports in the literature show that they can participate in the chelation of Cd. For instance, the PDF2 (AtPDF2.5) in Arabidopsis is involved in Cd tolerance and accumulation, and Cd binding analysis shows that AtPDF2.5 has Cd chelating activity in vitro [70]. Overexpression of AtPDF2.6 increases the tolerance of *E. coli* and yeast to Cd, and the in vitro Cd binding test shows that AtPDF2.6 also has Cd chelating activity [71]. The PDF1.5 is located in the cell wall and may detoxify Cd by chelating Cd to the cell wall [61]. PDF has a characteristic three-dimensional folding pattern, which is stabilized by eight disulfide bonds between cysteines [72]. Cysteine and other key residues are necessary to induce changes in the three-dimensional structure of AtPDF, which can mediate and increase yeast tolerance to Cd [73].

### 3.3. Comparison of the Core Features of Key Cd Chelation Proteins

Analyzing the proteins involved in Cd chelation in the MT and PDF families showed that these proteins all have a cysteine-rich domain (Table 2). Cysteine is a sulfur-containing amino acid with physiological activity, with amino and sulfhydryl groups. Among protein amino acids, cysteine may be the only amino acid that contains a thiol group on the side chain and has a binding site for heavy metals of plant cells [74]. PDF can chelate Cd in the cell wall, thereby preventing Cd from destroying the cell structure, and has a more effective detoxification ability. Nevertheless, MT forms a chelate with Cd in the cytoplasm, and is then transferred to the vacuole by other transporters for detoxification. Interestingly, there is a special signal peptide at the N-terminus of the PDF protein, which may mediate its secretion in the cell wall. Surprisingly, OsMTI-1b and OsMT1e are expressed in the nucleus, indicating that members of the MT family seem to be involved in the regulation of gene transcription. On the whole, the cell wall is a unique cell structure of plants, and it is the first protective barrier to prevent heavy metals from invading cells. The specific signal peptide at the N-terminus of PDF protein has great research value for the genetic improvement of crop Cd tolerance, and how the MT family members regulate gene expression in the nucleus remains to be explored.

## 4. Vacuolar Protein

The cellular mechanism of Cd is generally considered to include isolation in the cytoplasm by glutathione, glutathione-related plant chelator, and isolation in the vacuole by the vacuolar membrane Cd/H antiporter. In fact, most plants detoxify heavy metals by storing Cd-chelating complexes or free Cd in vacuoles, because this organelle contains enzymes involved in Cd detoxification, such as phosphatase, lipase, and protease. The main proteins involved in the transfer of Cd into vacuoles in plants are CAX, ABCC and MTP family members.

### 4.1. Introduction to CAX Family, Functional Proteins and Vacuolar Transfer Mechanism

CAX is a secondary charged ion transporter located in the vacuole, which relies on the secondary energy transport of metal ions in the proton transmembrane gradient [79]. The main function of CAX protein is to limit the toxicity of certain free metal ions to cells, such as Mn or Cd, provide tolerance to metal toxicity, and maintain cytosolic concentration [80]. For example, the expression of AtCAX2 in tobacco increases the transport of Cd in root vacuoles [81]. AtCAX4 compensates to a certain extent for the reduction of the vacuolar membrane H pump and the increase in proton leakage, which helps to improve the higher tolerance of plants to Cd [82]. OsCAX1a/OsCAX1c may also be involved in the process of Cd transfer to vacuole storage [83]. In plants, CAX is driven by the pH gradient produced by vacuolar H-ATPase and H-pyrophosphatase to drive metal ion transport. Studies have shown that the local primary structure determines the cation specificity of CAX binding [84]. There is a highly conserved 36 motif region between TM3 and TM4, TM8 and TM9, named c-1 loop and c-2 loop, respectively, which may form a vestibule or filter for cation selection, and there are highly conserved GNXXE residues (Figure S5) [85]. CAX has a common motif, which is the region rich in acidic amino acid motifs between TM6 and TM7, which is believed to be related to the capture and selection of metal ions.

### 4.2. An Introduction to the ABCC Subfamily, Functional Proteins and Vacuolar Transfer Mechanism

The ABC transporter is one of the largest and oldest protein families; it plays a very crucial part in plant heavy metal transport and plant disease resistance. A total of 9 subfamilies (ABCA-ABCI) have been identified in eukaryotes, of which 8 subfamilies (ABCA-ABCG and ABCI) exist in the plant genome. In plants, the two subfamilies that are currently reported to be related to heavy metals are mainly ABCC and ABCG. ABCC mainly transfers Cd to vacuoles, while ABCG removes Cd from cells for detoxification. The AtABCC1 and AtABCC2 participate in the vacuolar separation of PC-Cd (II) and PC-Hg (II), thereby making plants tolerant to these toxic heavy metals [86]. AtABCC3 detoxifies by transporting the PC-Cd complex into the vacuole [87], and the OsABCC9 isolates Cd into the vacuoles of rice roots to mediate Cd tolerance and accumulation [88]. They are usually composed of two membrane domains or TMDs and two cytoplasmic nucleotide binding domains (NBD) [89]. Two TMDs provide channels for metal ions, while two NBDs combine and hydrolyze ATP to provide energy for transport reactions [90]. As the input proteins, TMD and NBD act as separate polypeptide chains, while in the bacterial export protein, a TMD and NBD are fused to form a homodimer or a heterodimer, thereby producing a complete and functional transport protein [91]. In particular, high-resolution electron microscopy has determined the structure of 8 different states and conformations, including two inward (IF), four outward (OF) and two asymmetric hydrolyzed states [92]. A single conformational transition from IF to OF conformation, triggered by ATP binding, drives unidirectional substrate translocation across the membrane. After this power stroke, ATP hydrolysis and phosphate release start to return to a static state, which promotes nucleotide exchange and a new round of substrate binding and translocation [93].

### 4.3. Introduction to MTP Family, Functional Proteins and Vacuolar Transfer Mechanism

CDF is also known as MTP in plants. MTP/CDF is a type of membrane-bound protein, which can maintain cell homeostasis and play a vital part in the process of plants dealing with heavy metal stress. It is a ubiquitous divalent cation transporter and is essential for the metal homeostasis and tolerance of archaea, bacteria and eukaryotes. Most of the CDF family members confer heavy metal tolerance by affecting the outflow of heavy metals from the cytoplasm. Due to the different members, outflow refers to the outflow from the cell, or into the internal compartment [94,95]. In the model plants rice and Arabidopsis, members of the MTP family mainly transfer heavy metals into vacuoles to increase plant tolerance. For instance, heterologous expression of OsMTP1 in tobacco plants increases the plant’s tolerance to Cd toxicity and allows transgenic plants to accumulate more Cd [96]. After 24 h of Cd stress, the expression levels of OsMTP6, OsMTP7, OsMTP9 and MTP11.1 in the shoots of rice increased significantly [90], but there is no research showing how they respond to Cd stress [97]. All CDF transporters consist of two domains: the TMD for cation transport and the regulatory cytoplasmic C-terminal domain (CTD) [98]. The combination of metal ions and CTD will induce its dimerization, and it will lead to a higher CTD accumulation [99]. Although CTD shows a high degree of sequence variability between different species, all available CTD structures have similar metal chaperone-like folds [100]. Metal chaperones are cytoplasmic metal carrier proteins that form a metal donor–acceptor interface with related transport mechanisms to transport metal ions to various protein targets and participate in cytoplasmic metal transport [101]. They usually have 6 TMDs and have a highly conserved characteristic sequence in TMD 2 and TMD 5, which has a potential effect on metal selectivity [102,103]. There are also HXXXD or DXXXD conserved residues on or near these 2 TMDs. If this residue is subjected to site-directed mutation, the CDF transporter will lose its ability to bind to Cd and impair Cd transport [104]. However, so far, the metal selectivity of the substrate among members of the CDF family is still unclear. We speculate that these two conserved and aspartic acid-rich residues are key targets for the binding and transport of metal ions. Furthermore, some MTP transporters have an (HX)n between TMD 4 and TMD 5 that also exists in members of the ZIP family and has similar functions. It can be used as a Zn buffer bag and a cytoplasmic surface zinc level that recognizes Sensor [105].

### 4.4. Comparison of Core Characteristics of Key Proteins in Vacuolar Transport

Vacuoles are the largest organelles in mature plant cells. They are a reservoir of ions and metabolites and are indispensable for the detoxification process and normal cell development. By comparing the results of subcellular localization, it is found that CAX/MTP/ABCC family members are mainly located on the vacuole membrane, which will increase the content of Cd in overexpressed plants, indicating that these proteins are detoxified by transferring Cd to the vacuole storage (Table 3). H/DXXXD residues are widely distributed in these three family members (Figures S5 and S6), whether they are a specific Cd binding site remains to be considered. The difference is that CAX and ABCC family members can be expressed both in the vacuole membrane and in the vacuole. Whether they have multiple functions, such as chelating Cd in the vacuole and transporting on the vacuole membrane, remains to be studied in depth. The ABCC transporter in particular can not only transport Cd directly, but also transport the PC-Cd complex. Whether there is a specific theme to give this interesting substrate difference or not requires continued research.

## 5. Long-Distance Transport Protein

In the evolution of plants, with the increase in body size, specialized transport organization-vascular bundles gradually developed. Vascular bundles are composed of xylem and phloem, where the phloem plays a leading role in the long-distance transportation of Cd from root to leaf [106]. The transfer of Cd from root to shoot can be divided into two stages: transfer from xylem parenchyma cells to ducts and transport in ducts. The former is mainly loaded with Cd into the xylem duct by the transport protein on the vacuole membrane, which promotes the loading into the xylem. The transport capacity of the latter is affected by transpiration and root pressure. OPT and HMA are the main proteins involved in the long-distance transport of Cd in rice and Arabidopsis.

### 5.1. Introduction to the OPT Family, the Key Proteins Involved in the Transport of Cd and the Mode of Transport

Members of the OPT family were first characterized in yeast and subsequently found in archaea, bacteria, and plants [107]. Phylogenetic analysis revealed that OPT members in plants were divided into two subfamilies: the yellow striped (YSL) protein and the oligopeptide transporter PT [108]. Interestingly, only OPT was found to be able to transport metal composites over long distances instead of directly transporting metals [109]. The AtOPT6 in Arabidopsis may be involved in the transport of complexes with GSH, PC and Cd to sink organs [110,111]. The OPT family is a group of electrochemical potential-driven transport proteins that catalyze solutes in an energy-dependent co-transport mode. AtOPT4 is a H-coupled high-affinity transporter that can participate in the loading/unloading of Cd-GSH in siliques. The transport of tetrapeptides GGFM, GGFL and KLGL mediated by it is electrogenic [112,113]. The proton and the substrate are transported together in a stoichiometric manner, resulting in a net positive charge in each transport cycle, which is independent of the charge of the substrate. There are two highly conserved residues (NPG and KIPPR) in OPT members (Figure S7), but the current understanding of the functions of these two conserved residues is very limited [114].

### 5.2. An Introduction to the HMA Family, the Key Proteins Involved in the Transport of Cd and the Mode of Transport

HMA only exists in vascular plants and contributes a significant part to phytoremediation through long-distance transportation from roots to the above ground tissues. HMA is a P1B member of the ATPase family in plants, and has been extensively studied for its important role in phytoremediation [115]. This gene family is mainly divided into two categories: Cu/Ag (Cu-ATPase) and Zn/Co/Cd/Pb (Zn-ATPase), and molecular genetic studies have shown that HMA2, 3, and 4 are mainly involved in Zn/Cd transport [116]. The contents of zinc and Cd in branches of AtHMA4 overexpressed lines increased, suggesting that AtHMA4 plays a role in metal loading into the xylem [117]. The loss of AtHMA2 and AtHMA4 function leads to almost complete elimination of root-to-stem Cd translocation, indicating that this is the main mechanism of Cd translocation in Arabidopsis [118]. OsHMA2 contributes greatly to the transport of Zn and Cd from root to stem, and participates in the transport of Zn and Cd to rice seed development [119]. HMA uses energy from ATP hydrolysis to transport ions across the cell membrane. Their domains have different types, which may be a N-terminal extension, containing one or more CXXC residues, a C-terminal extension rich in metal-binding amino acids (Figure S8), or both [120]. These metal-binding domains have been shown to coordinate Zn and Cd in bacteria [121]. The functional studies show that the side chains of Methionine 254, Cysteine 476 and Histidine 807 of HMA in *Cupriavidus metallidurans* help Cd, Co and Zn binding and transport [122].

### 5.3. Comparison of Core Characteristics of Key Proteins for Long-Distance Transportation of Cd

We found that OPT is a protein that transports metal chelate indirectly, while HMA can transport metal ions directly, and they are widely present in the vascular tissues of plant roots, stems and leaves. A comparative analysis of the two family members found that the HMA family members are rich in cysteine residues, but OPT does not, indicating that this residue may be involved in the direct binding of Cd (Table 4). This may be a pivotal reason why HMA can transport heavy metal ions, while OPT basically transports heavy metal complexes. It is extraordinary that OsHMA2, like OsMTI-1b and OsMT1e, can be expressed in the nucleus, and they may be involved in gene transcription regulation. Based on these findings, if research on metal transporters and gene expression regulation can be carried out, it is expected to fill this gap.

## 6. Efflux Protein

After being stressed by heavy metals, the physiological and biochemical functions of plants will be affected to varying degrees. In order to maintain Cd within the physiological tolerance limit range of plants, plants will have a series of steady-state systems and potential mechanisms involved in the detoxification of heavy metals. One of the important mechanisms of Cd detoxification is to control the efflux of Cd from plants, reducing damage to plants. Generally, the ABCG and PCR family members are mainly involved in Cd efflux.

### 6.1. A Brief Introduction to the PCR Family, Key Efflux Proteins and Efflux Mechanisms 

PCR is a huge gene family, and a large number of members can be found in fungi, algae, higher plants and animals. They have the common cysteine-rich PLAC8 domain, which is reported to be related to the Cd resistance of plants [123]. Beyond that, CCXXXXCPC or CLXXXXCPC (X = any amino acid) residues were also discovered (Figure S9), and the CCXXXXCPC motifs may participate in the binding of divalent cations, and then complete the transfer of divalent cations [124,125]. Most proteins containing the PLAC8 domain have two transmembrane α-helices, indicating that they are membrane-associated proteins or membrane intrinsic proteins, and have two distinct functions of plants [126]. On the one hand, they participate in the determination of fruit size and cell number, on the other hand, they participate in the transport of heavy metals such as Cd or zinc [127,128]. Overexpression of AtPCR1 reduced the influx of Cd by yeast cells, and also reduced the Cd content of yeast and Arabidopsis protoplasts treated with Cd [129]. Overexpression of OsPCR1 and OsPCR3 not only improved the tolerance of transgenic rice to Cd, but also significantly reduced the accumulation of Cd in different parts of the transgenic rice (especially rice grains) [130]. Although they are widely distributed, our understanding of the functions and transport mechanisms of these proteins is very limited.

### 6.2. A Brief Introduction to the ABCG Family, Key Efflux Proteins and Efflux Mechanisms

ABCG is the largest subfamily of ABC transporters in plants; it is mainly divided into WBC and PDR. The PDR only exists in plants and fungi, they are the most numerous types. One hallmark of PDR transporters is their asymmetry, and the typical nucleotide binding site can undergo ATP hydrolysis, while the other cannot. It takes part in the influx, accumulation, defense and efflux process of toxic substances and metabolites. So far, except for the ABCG19 transporter, all the full-size and half-size ABCG transporters studied have been found to be located in the plasma membrane [131]. The AtABCG36 is mainly expressed in the epidermis of roots and leaves, and overexpression of this gene increases the resistance of plants to Cd and Pb, reduces the Cd content in plants, and is the main Cd efflux pump in the plasma membrane of Arabidopsis [132]. The heterologous expression of OsABCG36 in yeast shows Cd efflux activity, and knocking out this gene will increase the accumulation of Cd in the root cell sap of rice and can also enhance the sensitivity of plants to Cd stress [133]. 

### 6.3. Comparison of Core Features of Key Cd Efflux Proteins

The members of the PCR protein contain the CCXXXXCPC motif, while the members of the ABCG family involved in Cd efflux contain CXXC residues (Table 5, Figure S10). These cysteine-rich residues may be involved in the binding and transport of divalent cations. In the analysis of the primary structure of the proteins of the members of the PCR family, surprisingly, we found that these members are all membrane proteins, but do not contain a TMD. The PCR protein may excrete Cd ions to the extracellular space through exocytosis. There is no report on the mechanism of how PCRs complete the Cd efflux, and it remains to be further studied by botanists.

## 7. Conclusions and Outlook

**Figure 2:**
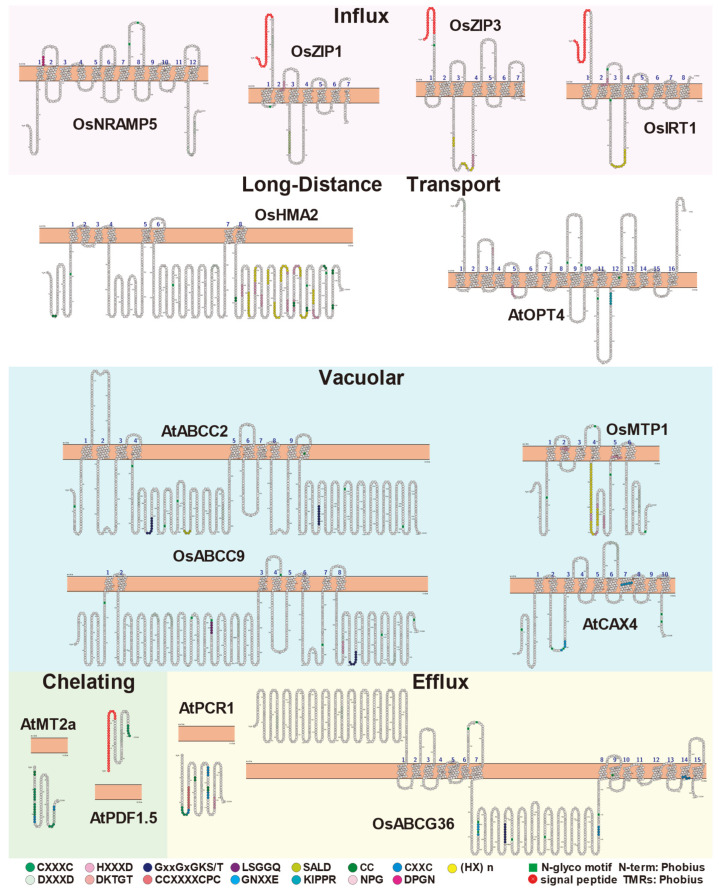
Residue-specific and transmembrane structural characterization of proteins. In each family, 1–2 special proteins involved in Cd influx, long-distance transport, vacuolar transport, chelating, and efflux were selected. Each type of protein is marked with a different colored box. Their specific residues and transmembrane structure characteristics were analyzed. The remaining member information can be found in Figures S1–S10, and their amino acid sequences are also summarized in Table S1. Different residues are indicated by different colors. The amino acid composition of the residue is indicated by the abbreviated letter of the amino acid in the circle. Yellow rectangles represent biofilms, green boxes represent N-glycan motifs, and red circles and white font represent signal peptides.

Through the comparative analysis of the metalloproteins involved in Cd influx, transport and efflux, we found that their mechanism of action is different. Their specific motifs and transmembrane structural characteristics are summarized in Figure 2. At the same time, we also summarized the key motifs based on the conservative or unique key residues of these different metalloproteins (Table 6). Based on these findings, further research combined with experiments will facilitate the understanding of the interaction mechanism between Cd-protein molecules.

Generally, proteins containing cysteine residues (such as CC, CXXC, CXXXC) can bind or transport Cd. The AtOPT4 lacking these residues can only transport Cd complexes, indicating that this motif may be a key residue involved in the direct interaction of Cd. H/DXXXD residues are widely present in almost all Cd transporters, and they may play an indispensable role in Cd transport and detoxification. Different motifs: We have summarized the residues that exist independently in each family, such as NPG, KIPPR and GNXXE, but it is still unclear whether residues are only involved in the composition of the protein structure or are related to the binding of Cd. In addition, HXXXD, DXXXD, CXXXC, CXXC, CC, (Hn)x motifs are widely present in the metalloprotein family, but is the difference in motifs related to the binding ability of Cd? Which of the above motifs plays a major role in binding to Cd remains to be studied. Interestingly, some members of the ZIP and PDF families may undergo large-scale expansion under environmental pressure, causing the emergence of N-terminal specific signal peptides and the diversified functions. For example, AtPDF1.5 is specifically secreted in the cell wall, chelating Cd in the cell wall, or inducing the expression of OsZIP3 in xylem parenchyma cells, transporting heavy metals to the xylem. These gene mutations endow plants with excellent Cd detoxification ability, so the design and breeding based on these signal peptides are expected to become a research hotspot in the future. Surprisingly, both the long-distance transport protein OsHMA2 and the chelating protein OsMT1e can be expressed in the nucleus. Are they also involved in directing gene transcription regulation? The emergence of multiple functions means their particularity, which opens up new directions for future research.

In summary, the metalloproteins we have summarized can be used as research objects for crystallography and molecular intelligence breeding. It is expected to provide certain guiding options for understanding the Cd–protein interaction mechanism and provide a basis for the creation of Cd-tolerant crops.

## Figures and Tables

**Table 1:** Summary of tissue structure-specific expression, structural characteristics and functions of key Cd influx proteins.

The Key Proteins	Tissue Specific Expression	Subcellular Localization	Structural Features	References
AtNRAMP1	Mainly expressed in the root	Cell membrane	12 TMDs, DPGN near to TMD1.	[53,54]
OsNRAMP1	Mainly expressed in the mature zone of the root	Cell membrane	12 TMDs, DPGN near to TMD1.	[32,55]
OsNRAMP5	Mainly expressed in the root	Cell membrane	10 TMDs, DPGN is near to TMD1. There is a Asparticacid residue (DXXXD, X= any amino acid) at the C-terminus.	[31]
AtIRT1	Root epidermal cells	Cell membrane	8 TMDs; there is a signal peptide at the N-terminus, histidine, Asparticacid residues (HXXXD, X= any amino acid) on TMD2, and (HX)n between TMD3 and TMD4.	[40]
OsIRT1	Mainly expressed in the root	Cell membrane	8 TMDs; there is a signal peptide at the N-terminus, HXXXD residues on TMD2, and the (HX)n residues between TMD3 and TMD4.	[56]
OsIRT2	Mainly expressed in the root	Cell membrane	There are 8 TMDs with signal peptide and DXXXD residues at the N-terminus; the (HX)n residues between TMD3-TMD4.	[56]
OsZIP1	Mainly expressed in the root	Cell membrane	8 TMDs, with signal peptide and DXXXD residues at the N-terminus; HXXXD residues on TMD2, and the (HX)n residues between TMD3-TMD4.	[41]
OsZIP3	Xylem parenchyma cells	Cell membrane	There are 7 TMDs, there is a signal peptide at the N-terminus, and there are (HX)n residues and HXXXD conserved residues between TMD3-TMD4.	[41,57]

**Table 2:** The tissue structure specific expression, structure characteristics and function summary of the key proteins of Cd chelation.

The Key Proteins	Tissue Specific Expression	Subcellular Localization	Structural Features	References
AtMT2a	/	Cytoplasm	CXCXXXCXC and DXXXD are distributed at the C end, without TMDs.	[75]
AtMT3	/	Cytoplasm	CXCXXXCXCXXXCXC is distributed at the C-terminus and has no TMDs.	[75,76]
OsMTI-1b	/	Cytoplasm, Nucleus	CXCXXXCXCXXXCXC is distributed at the C-terminus and has no TMDs.	[62]
OsMTI-2b	Highly expressed in rice stems	Cytoplasm	N-terminal has 8 cysteine residues arranged in CC, CXC and CXXC residues.	[77]
OsMT1e	Expressed in roots at all developmental stages	Nucleus	CXCXXXCXCXXCXCXCXCX is distributed at the N-terminal and has no TMDs.	[78]
OsMT-3a	/	Cytoplasm	CXCXXXCXCXXCXCXCXCX is distributed at the C-terminus and has no TMDs.	[59,64]
AtPDF1.5	Mainly expressed in knots and peels	Cell wall and cytoplasm	There is a signal peptide at the N-terminus, a cysteine-rich domain, and a DXXXD residue.	[72]
AtPDF2.5	mainly expressed in root vascular bundle	Cell wall	Cysteine-rich domain and secretion signal peptide.	[70]
AtPDF2.6	mainly expressed in root vascular bundle	Cell wall	There is a signal peptide at the N-terminus, a cysteine-rich domain.	[71]

**Table 3:** Tissue structure-specific expression, structural characteristics and function summary of key proteins in vacuolar transport.

The Key Proteins	Tissue Specific Expression	Subcellular Localization	Structural Features	References	
AtCAX2	/	Tonoplast	8 TMDs; with GNXXE residue on TMD2 and TMD7.	[81]	
AtCAX4	Mainly expressed in the root	Tonoplast	10 TMDs; a GNXXE residue on TMD7, and the DXXXD residues between TMD5-6.	[82]	
OsCAX1a	expressed at a high level in flowering spikelet	Vacuole	10 TMDs; GNXXE residues on TMD3 and TMD8, and the DXXXD residues between TMD6-TMD7.	[83]	
OsCAX1c	Strongly expressed in leaves	Tonoplast	10 TMDs; GNXXE residues exist on TMD2, TMD, and DXXXD residues near TMD6.	[83]	
AtABCC1	Mainly expressed in the root	Vacuole	15 TMDs, nucleotide binding site at the C-terminus, cysteine-rich loop, Q-loop, Walker B residue.	[86]	
AtABCC2	Mainly expressed in the root	Vacuole	10 TMDs, cysteine-rich loop, Walker B residue, DXXXD residues exist between TMD4-TMD5.	[86]	
AtABCC3	/	Tonoplast	15 TMDs, cysteine-rich loop, Walker B residue, HXXXD residues near TMD3, and DXXXD residues between TMD9-TMD10.	[87]	
OsABCC9	Significantly induced by Cd treatment in roots	Tonoplast	8 TMDs, cysteine-rich loop, Q-loop, Walker B residue, and HXXXD and DXXXD residues at the N-terminus.	[88]	
OsMTP1	Significantly induced by Cd treatment in roots	Vacuole	The characteristic residue of CDF (SLAILTDAAHLLSDVAA), 6 TMDs and histidine-rich regions, HXXXD residues exist on and/or near TMD2, TMD5.	[96]	

**Table 4:** Structure specific expression, structure characteristics and function summary of key proteins for Cd long-distance transport.

The Key Proteins	Tissue Specific Expression	Subcellular Localization	Structural Features	References
AtOPT6	It is preferentially expressed in the vascular system of rosette leaves, stems and roots	Cell membrane	15 TMDs, including NPG (near TMD3), KIPPR (near TMD12) residues, HXXXD residues near TMD5, and DXXXD residues at the N-terminal.	[110,111]
AtOPT4	Mainly expressed in the vascular bundles	Cell membrane	14 TMDs, including NPG (tMD2-TMD3), KIPPR (TMD10-TMD11) residues.	[112,113]
AtHMA4	Mainly expressed in the vascular tissues of roots, stems and leaves	Cell membrane	There are 6 CC pairs, (HX)n, DXXXD residues and two repeats of the GXDSGCCGXKSQQPHQHEXQ sequence at the C-terminus.	[117,118]
AtHMA2	Mainly expressed in the vascular tissues of roots, stems and leaves	Cell membrane	8 TMDs, an extended C-terminus, potential metal binding residues (13 cysteine pairs and 11 histidine sequences).	[118]
OsHMA2	Root	Cell membrane, Nucleus	8 TMDs, CC pairs, (HX)n, HXXXD residues are widely distributed at the C-terminus.	[119]

**Table 5:** Summary of tissue structure-specific expression, structural characteristics and functions of key Cd efflux proteins.

The Key Proteins	Tissue Specific Expression	Subcellular Localization	Structural Features	References
AtPCR1	Expressed in all organizations	Plasma membrane	No TMDs, α-membrane protein helix, CCXXXXCPC motif, HXXXD residue	[129]
OsPCR1	Mainly expressed in root of seedling and internode I and II of reproductive stage, but also in spikelet	Plasma membrane	No TMDs, including CCXXXXCPC motif, HXXXD residue	[130,134]
OsPCR3	Root	Plasma membrane	No TMDs, a CCXXXXCPC residue	[130]
AtABCG36	The expression level was highest in root hair and epidermal cells	Plasma membrane	15 TMDs, including GxxGxGKS/T and DXXXD residues between TMD7-TMD8	[132]
OsABCG36	It was highly expressed in roots under Cd stress	Plasma membrane	15 TMDs with DXXXD residues at the N-terminus, GxxGxGKS/T, GNXXE, CXXC residues were found between and TMD7-TMD8, CXXC residues were found on TMD14	[133]

**Table 6:** Summary of Proteins with Value for Continued Research.

Protein ID	Function Type	Tissue Specific	Summary of Metalloproteins with Research Value
OsNRAMP5	Influx protein	Cell membrane in roots	The main Cd uptake and transporter in rice; DPGN motis are only found in NRAMP.
OsZIP3	Influx protein	Cell membrane of xylem parenchyma	The appearance of the N-terminal specific signal peptide may induce its expression in xylem parenchyma cells and diversify the functions of members of the ZIP family.
AtPDF1.5	Chelating protein	Cell wall and cytoplasm in nodes and peels	The N-terminal signal peptide may mediate its secretion in the cell wall, chelate Cd in the cell wall, and is rich in cysteine-related motifs.
OsMT1e	Chelating protein	Nucleus in roots	Expressed in the nucleus, indicating that members of the MT family seem to be involved in the regulation of gene transcription, and are rich in cysteine-related motifs.
AtOPT4	Long-distance transport protein	Cell membrane in vascular bundle	Participate in the long-distance transport of the GSH-Cd complex, no residues related to cysteine, including NPG and KIPPR motifs unique to this family.
OsHMA2	Long-distance transport protein	Cell membrane and Nucleus in the roots	Participate in the long-distance transportation of Cd, which may be because the C-terminal cysteine-rich motifs can directly bind to Cd. Expressed in the nucleus, they may be involved in gene transcription regulation.
AtPCR1	Efflux protein	Plasma membrane of all tissues	It is a membrane protein but does not contain a TMD. It may excrete Cd ions to the extracellular space through exocytosis, and contains CCXXXXCPC motifs.
OsABCG36	Efflux protein	Plasma membrane of roots	It is highly expressed in roots under Cd stress and contains motifs related to cysteine. Exporting Cd or Cd conjugates.

## Data Availability

Not applicable.

## Supplementary Materials

The following are available online at https://www.mdpi.com/article/10.3390/ijms23031734/s1.

## Abbreviations

ABA	abscisic acid
ABC	ATP-binding cassette transporters
At	*Arabidopsis thaliana*
Bb	*Bordetella bronchiseptica*
CAX	Cation/proton exchanger
Cd	cadmium
HMA	heavy metal ATPase
IRT1	iron-regulated transporter 1
JA	jasmonic acid
MT	metallothionei
MTP/CDF	metal tolerance protein/ cation diffusion facilitator
NRAMP	Natural resistance-associated macrophage protein
OPT	oligopeptide transporter
Os	*Oryza sativa*
PCR	plant Cd resistance
PDF	plant defensins
SA	salicylic acid
TFs	transcription factor
ZIP	ZRT, IRT-like protein
