# Supplementary material for: Metalloprotein-Specific or Critical Amino Acid Residues: Perspectives on Plant-Precise Detoxification and Recognition Mechanisms under Cadmium Stress
Source: Int J Mol Sci. 2022 Feb 3;23(3):1734. doi: 10.3390/ijms23031734 (PMC8836122; doi:10.3390/ijms23031734)
Supplement: Supplementary file 1 [file ijms-23-01734-s001.zip › ijms-1558906-SI.pdf]

SUPPLEMENTARY MATERIALS

Metalloprotein-specific or critical amino acid residues: Perspectives on plant-precise detoxification and recognition mechanisms under cadmium stress

Dandan Li [1], Tengbing He [1,2], Muhammad Saleem [3], and Guandi He [1,*]

1 College of Agricultural, Guizhou University, Guiyang 550025, Peoples R China; gs.lidd19@gzu.edu.cn (D.L.); tbhe@gzu.edu.cn (T.H.)

2 Institute of New Rural Development, West campus, Guizhou University, Guiyang 550025, China

3 Department of Biological Sciences, Alabama State University, Montgomery, AL 36104 USA; msaleem@alasu.edu (M.S.)

* Correspondence: gdhe@gzu.edu.cn (G.H.)

Table S1: Functional protein sequence involved in Cd absorption, transport and detoxification.

Figure S1: Specific residues and transmembrane structural features of NRAMP proteins involved in Cd influx.

Figure S2: Specific residues and transmembrane structural features of ZIP proteins involved in Cd influx.

Figure S3: Specific residues and transmembrane structural features of MT proteins involved in Cd chelation.

Figure S4: Specific residues and transmembrane structural features of PDF proteins involved in Cd chelation.

Figure S5: The specific residues and transmembrane structural features of the vacuolar proteins in CAX, MTP family members.

Figure S6: The specific residues and transmembrane structural features of the vacuolar proteins in ABCC family members.

Figure S7: Specific residues and transmembrane structural features in the OPT family of long-distance transport proteins.

Figure S8: Specific residues and transmembrane structural features in the HMA family of long-distance transport proteins.

Figure S9: Specific residues and transmembrane structural features of members in PCR the family of efflux proteins.

Figure S10: Specific residues and transmembrane structural features of members in the ABCG family of efflux proteins.

Table S1: Functional protein sequence involved in Cd absorption, transport and detoxification.

>AtNRAMP1

MAATGSGRSQFISSSGGNRSFSNSPLIENSDSNQIIVSEKKSWKNFFAYLGPGFLVSIAYIDPGNFETDLQAGAHYKYELLWIILVASCAALVIQSLAANLGVVTGKHLAEQCRAEY
SKVPNFMLWVVAEIAVVACDIPEVIGTAFALNMLFSIPVWIGVLLTGLSTLILLALQKYGVRKLEFLIAFLVFTIAICFFVELHYSKPDPGEVLHGLFVPQLKGNGATGLAISLLGAM
VMPHNLFLHSALVLSRKIPRSASGIKEACRFYLIESGLALMVAFLINVSVISVSGAVCNAPNLSPEDRANCEDLDLNKASFLLRNVVGKWSSKLFAIALLASGQSSTITGTYAGQYV
MQGFLDLRLEPWLRNLLTRCLAIIPSLIVALIGGSAGAGKLIIIASMILSFELPFALVPLLKFTSCKTKMGSHVNPMAITALTWVIGGLIMGINIYYLVSSFIKLLIHSHMKLILVVFCGI
LGFAGIALYLAAIAYLVFRKNRVATSLLISRDSQNVETLPRQDIVNMQLPCRVSTSDVD

>OsNRAMP1

MGVTKAEAVAGDGGKVVDDIEALADLRKEPAWKRFLSHIGPGFMVCLAYLDPGNMETDLQAGANHKYELLWVILIGLIFALIIQSLSANLGVVTGRHLAELCKTEYPVWVKTC
LWLLAELAVIASDIPEVIGTGFAFNLLFHIPVWTGVLIAGSSTLLLLGLQRYGVRKLEVVVALLVFVMAGCFFVEMSIVKPPVNEVLQGLFIPRLSGPGATGDSIALLGALVMPHNL
FLHSALVLSRNTPASAKGMKDVCRFFLFESGIALFVALLVNIAIISVSGTVCNATNLSPEDAVKCSDLTLDSSSFLLRNVLGKSSATVYGVALLASGQSSTITGTYAGQYVMQGFL
DIKMKQWLRNLMTRSIAIVPSLIVSIIGGSSGAGRLIVIASMILSFELPFALIPLLKFSSSSNKMGENKNSIYIVGFSWVLGFVIIGINIYFLSTKLVGWILHNALPTFANVLIGIVLFPLM
LLYVVAVIYLTFRKDTVKFVSRRELQAGDDTEKAQVATCVADEHSKEPPV

>OsNRAMP5

MEIERESSERGSISWRASAAHDQDAKKLDADDQLLMKEPAWKRFLAHVGPGFMVSLAYLDPGNLETDLQAGANHRYELLWVILIGLIFALIIQSLAANLGVVTGRHLAEICKSEY
PKFVKIFLWLLAELAVIAADIPEVIGTAFAFNILFHIPVWVGVLITGTSTLLLLGLQKYGVRKLEFLISMLVFVMAACFFGELSIVKPPAKEVMKGLFIPRLNGDGATADAIALLGAL
VMPHNLFLHSALVLSRKTPASVRGIKDGCRFFLYESGFALFVALLINIAVVSVSGTACSSANLSQEDADKCANLSLDTSSFLLKNVLGKSSAIVYGVALLASGQSSTITGTYAGQYI
MQGFLDIRMRKWLRNLMTRTIAIAPSLIVSIIGGSRGAGRLIIIASMILSFELPFALIPLLKFSSSKSKMGPHKNSIYIIVFSWFLGLLIIGINMYFLSTSFVGWLIHNDLPKYANVLVGA
AVFPFMLVYIVAVVYLTIRKDSVVTFVADSSLAAVVDAEKADAGDLAVDDDEPLPYRDDLADIPLPR

>AtIRT1

MASNSALLMKTIFLVLIFVSFAISPATSTAPEECGSESANPCVNKAKALPLKVIAIFVILIASMIGVGAPLFSRNVSFLQPDGNIFTIIKCFASGIILGTGFMHVLPDSFEMLSSICLEENP
WHKFPFSGFLAMLSGLITLAIDSMATSLYTSKNAVGIMPHGHGHGHGPANDVTLPIKEDDSSNAQLLRYRVIAMVLELGIIVHSVVIGLSLGATSDTCTIKGLIAALCFHQMFEGM
GLGGCILQAEYTNMKKFVMAFFFAVTTPFGIALGIALSTVYQDNSPKALITVGLLNACSAGLLIYMALVDLLAAEFMGPKLQGSIKMQFKCLIAALLGCGGMSIIAKWA

>OsIRT1

MATPRTLVPILPPVAALLLLLVAASSIPILAAAQPADACGGAPDQAAADGACHDVPRALRLKLIAIPTILVSSVVGVCLPLLSRSVPALRPDGGLFAVVKAFASGVILATGYMHVLP
DAFNNLTSPCLPRKPWSEFPFAAFVAMLAAVSTLMADSLMLTYYNRSKPRPSSGGDVAAVADHGESPDQGHRHGHGHGHGHGMAVAKPDDVEATQVQLRRNRVVVQVLEIG
IVVHSVVIGLGMGASQNVCTIRPLVAAMCFHQMFEGMGLGGCILQAEYGRRMRSVLVFFFSTTTPFGIALGLALTRVYRDNSPTALIVVGLLNAASAGLLHYMALVELLAADFM
GPKLQGNVRLQLAAFLAVLLGAGGMSVMAKWA

>OsIRT2

MMMSSSQTPVRIAFVFLVILAATDAHSDHRTPPPACGGAAVGGECHSVARALRLKLIAIPAILAASVAGVCLPLFARSVPALRPDGGLFAVVKAFASGVILGTGYMHVLPDSFND
LTSPCLPRKPWSEFPFAAFVAMLAAVFTLMVDSLMLTFHTRGSKGRASSAVAHHGDHGHCHAHALGQADVAALSTTEAADQGSGDVEAGNTTKAQLLRNRVIVQVLEMGIVV
HSVVIGLGMGASQNVCTIRPLVAALCFHQMFEGMGLGGCILQAGYGGRTRSALVFFFSTTTPFGIALGLALTRVYSDSSPTALVVVGLLNAASAGLLHYMALVELLAADFMGPK
LQGNVRLQLAASLAILLGAGGMSVMAKWA

>OsZIP1

MARTMTMRVSSLLVAVVLLAALSFQACSGHGGINDGDGQVDAPATPASSSGVRSKGLIAVKVWCLVILLVFTFAGGVSPYFYRWNESFLLLGTQFAAGVFLGTALMHFLADST
STFKGLTTNQYPFSFMLTCVGFLLTMLSDLVIAAVARRSAAAGVSDNQVSEQQQRQQAEGAVMSRKEEEAAAVAHPAMLVRTSSFEDAVLLIVALCFHSVFEGIAIGVSASKSE
AWRNLWTIGLHKIFAAVAMGIALLRMIPKRPFLMTVVYSLAFAVSSPVGVGIGIAIDATSQGRAADWTYAISMGLATGVFIYVAINHLIAKGYRPHHPTAADKPLFKFLAVLLGV
[illegible]

>OsZIP3

MGAKKHTLQVLPWLLLFAQHTAASACDCANTTDGADRQGAMKLKLIAIASILAAGAAGVLVPVIGRSMAALRPDGDIFFAVKAFAAGVILATGMVHILPAAFDALTSPCLKRG
GGDRNPFPFAGLVSMSAAVSTMVVDSLAAGYYHRSQFRKARPVDNINVHKHAGDERAEHAQHINAHTHGGHTHSHGDIVVCGSPEEGSVAESIRHKVVSQVLELGILVHSVIIG
VSLGASVRPSTIRPLVGALSFHQFFEGVGLGGCIVQANFKVRATVIMAIFFSLTAPVGIVLGIAISSSYNVHSSTAFVVEGVFNSASAGILIYMSLVDLLATDFNNPKLQINTKLQLM
AYLALFLGAGLMSMLAIWA

>AtMT2a

MSCCGGNCGCGSGCKCGNGCGGCKMYPDLGFSGETTTTETFVLGVAPAMKNQYEASGESNNAENDACKCGSDCKCDPCTCK

>AtMT3

MSSNCGSCDCADKTQCVKKGTSYTFDIVETQESYKEAMIMDVGAEENNANCKCKCGSSCSCVNCTCCPN

>OsMTI-1b

MSCSCGSSCGCGSNCTCGKMYPDLEEKSSSAQATVVLGVAPEKAHFEAAAESGETAHGCGCGSSCKCNPCNC

>OsMTI-2b

MSCCGGNCGCGSSCQCGNGCGGCKYSEVEPTTTTTFLADATNKGSGAASGGSEMGAENGSCGCNTCKCGTSCGCSCCNCN

>OsMT1e

MSCSCGSSCSCGSNCSCGKKYPDLEEKSSSTKATVVLGVAPEKKAQQFEAAAESGETAHGCSCGSSCRCNPCNC

>OSMT-3a

MSDKCGNCDCADKSQCVKKGTSYGVVIVEAEKSHFEEVAAGEENGGCKCGTSCSCTDCKCGK

>AtPDF1.5

MAKFCTTITLILVALVLFADFEAPTIVKAELCKRESETWSGRCVNDYQCRDHCINNDRGNDGYCAGGYPW
YRSCFCFFSC

>AtPDF2.5

MENKFFAAFFLLLVLFSSQEIIGGEGRTCQSKSHHFKYMCTSNHNCAIVCRNEGFSGGRCHGFHRRCYCTRLC

>AtPDF2.6

MKLSLRLISALLMSVMLLFATGMGPVEARTCESPSNKFQGVCLNSQSCAKACPSEGFSGGRCSSLRCYCSKAC

>AtCAX2

MSCCKVPVLIEAQVEMVSANELENKSLFRQEEDATQTKEASLMEQGSLSTSFPQHTPKAPKNSVLNSIKIVIFCNKLNLLLPFGPLAILVHYMIDSKGWVFLLTLVGITPLAERLGY
ATEQLACYTGPTVGGLLNATFGNVTELIISIFALKNGMIRVVQLTLLGSILSNMLLVLGCAFFCGGLVFYQKDQVFDKGIATVNSGLLLMAVMGILFPAVLHYTHSEVHAGSSELA
LSRFSSCIMLIAYAAYLFFQLKSQSNSYSPLDEESNQNEETSAEDEDPEISKWEAIIWLSILTAWVSLLSGYLVDAIEGASVSWNIPIAFISTILLPIVGNAAEHAGAIMFAMKDKLDLS
LGVAIGSSIQISMFAVPFCVVIGWMMGQQMDLNFQLFETAMLFITVIVVAFFLQEGSSNYFKGLMLILCYLIVAASFFVHEDPHQDGI

>AtCAX4

MSSISTESSSNLSLLENGGGGSDKPTAETSRRVRRTVSASSLIRKRSDLKLISRVRWEFMRRILTNLQEVLLGTKLFILFPAVPLAVVAHRYDCPRAWVFALSLLGLTPLAERISFLT
EQIAFHTGPTVGGLMNATCGNATEMIIAILAVGQRKMRIVKLSLLGSILSNLLFVLGTSLFLGGISNLRKHQSFDPRQGDMNSMLLYLALLCQTLPMIMRFTMEAEEYDGSDVVV
LSRASSFVMLIAYLAFLIFHLFSSHLSPPPPPLPQREDVHDDDVSDKEEEGAVIGMWSAIFWLIIMTLLVALLSDYLVSTIQDAADSWGLSVGFIGIILLPIVGNAAEHAGAVIFAFRN
KLDITLGIALGSATQIALFVVPVTVLVAWTMGIEMDLNFNLLETACFALSILVTSLVLQDGTSNYMKGLVLLLCYVVIAACFFVSNSPSTETNTTNHTITKR

>OsCAX1a

MEAAAAMEAGRKLAARHPHGRSRTAHNMSSSSLRKKSDAALVRKVPVAPLRPLLANLQEVFLATKLAVLFPAVPLAIAAQCFRFDQVWVFALSLLGLIPLAERVSFLTEQIALY
TGPTVGGLLNATCGNATELIIALFALLKGKIEVVKCSLLGSVLSNLLLVLGTSLFCGGVVNLGARQPYDRNQSDVSTALLFLAVLCHSAPLLLRYAVAAGEHSVSATSAAASLDL
SRACSFVMLASYVAYLFFQLKTHRQLFEPQEVDGGDAGDDDEEPALGFASALFWLALMTAVISVLSEYVVGTIEPTSQSWGLSVSFISIILLPIVGNAAEHAGAIIFALKNKLDITLG
VALGSATQISMFVVPLSVLVAWIMGVQMDLDFKLLETGSLFMAVLVTAFTLQDGTSHYLKGILLLLCYIVIGACFFVARQPAGHANSNGALLDVPTGSMSVQA

>OsCAX1c

MAPPESSHHHLLESGLLEVSKAPSAAVAAEEEEKKEAAAWTPSSSSSMTGRKIKSEASPLLRRLLGGPAAQLQEVLLGTKLYPLFSAVPLAVAAESLRLGRVWVFAFSLIGLAPLA
ERVSFLSEHIANTVGPTAGGIMNATCGNVPELIIALFALHKNKMEILKWSLLGSILSNLLLVLGSSLLFGGIVNIGKERPLDKRQADVSIGLLLLGVLCHIATLVSKYTSSTGDSINSS
SVMQLSRSCAIVMLIAYFGSLMFQLKTHRQIFELEEDSSDSSSSEDDATDKSVIGFASAMVWLIGMAVVTAMLSSYVVTTIEEASESMGIPVRFISIILLPIVGNAAEHAGAIIFAFKN
KIDISLGITLGSATQISMLVVPVILIVSWVNAIPMDLDFNLLETGSLAMAVITTAFTLQDDKWHYLKGLNLVFSYIVIAVCFFVMKALPTLKKEDD

>AtABCC1

MGFEPLDWYCKPVPNGVWTKTVDYAFGAYTPCAIDSFVLGISHLVLLILCLYRLWLITKDHKVDKFCLRSKWFSYFLALLAAYATAEPLFRLVMRISVLDLDGAGFPPYEAFML
VLEAFAWGSALVMTVVETKTYIHELRWYVRFAVIYALVGDMVLLNLVLSVKEYYGSFKLYLYISEVAVQVAFGTLLFVYFPNLDPYPGYTPVGTENSEDYEYEELPGGENICPE
RHANLFDSIFFSWLNPLMTLGSKRPLTEKDVWHLDTWDKTETLMRSFQKSWDKELEKPKPWLLRALNNSLGGRFWWGGFWKIGNDCSQFVGPLLLNELLKSMQLNEPAWIGY
IYAISIFVGVVLGVLCEAQYFQNVMRVGYRLRSALIAAVFRKSLRLTNEGRKKFQTGKITNLMTTDAESLQQICQSLHTMWSAPFRIIVALVLLYQQLGVASIIGALFLVLMFPIQT
VIISKTQKLTKEGLQRTDKRIGLMNEVLAAMDTVKCYAWENSFQSKVQTVRDDELSWFRKAQLLSAFNMFILNSIPVLVTVVSFGVFSLLGGDLTPARAFTSLSLFSVLRFPLFM
LPNIITQMVNANVSLNRLEEVLSTEERVLLPNPPIEPGQPAISIRNGYFSWDSKADRPTLSNINLDIPLGSLVAVVGSTGEGKTSLISAMLGELPARSDATVTLRGSVAYVPQVSWIF
NATVRDNILFGAPFDQEKYERVIDVTALQHDLELLPGGDLTEIGERGVNISGGQKQRVSMARAVYSNSDVCILDDPLSALDAHVGQQVFEKCIKRELGQTTRVLVTNQLHFLSQV
DKILLVHEGTVKEEGTYEELCHSGPLFQRLMENAGKVEDYSEENGEAEVDQTSVKPVENGNANNLQKDGIETKNSKEGNSVLVKREERETGVVSWKVLERYQNALGGAWVV
MMLVICYVLTQVFRVSSSTWLSEWTDSGTPKTHGPLFYNIVYALLSFGQVSVTLINSYWLIMSSLYAAKKMHDAMLGSILRAPMVFFQTNPLGRIINRFAKDMGDIDRTVAVFV
NMFMGSIAQLLSTVILIGIVSTLSLWAIMPLLVVFYGAYLYYQNTSREIKRMDSTTRSPVYAQFGEALNGLSSIRAYKAYDRMAEINGRSMDNNIRFTLVNMAANRWLGIRLEVL
GGLMVWLTASLAVMQNGKAANQQAYASTMGLLLSYALSITSSLTAVLRLASLAENSLNSVERVGNYIEIPSEAPLVIENNRPPPGWPSSGSIKFEDVVLRYRPELPPVLHGVSFLIS
PMDKVGIVGRTGAGKSSLLNALFRIVELEKGRILIDECDIGRFGLMDLRKVLGIIPQAPVLFSGTVRFNLDPFSEHNDADLWESLERAHLKDTIRRNPLGLDAEVTEAGENFSVGQR
QLLSLARALLRRSKILVLDEATAAVDVRTDVLIQKTIREEFKSCTMLIIAHRLNTIIDCDKVLVLDSGKVQEFSSPENLLSNGESSFSKMVQSTGTANAEYLRSITLENKRTREANGD
DSQPLEGQRKWQASSRWAAAAQFALAVSLTSSHNDLQSLEIEDDNSILKKTKDAVVTLRSVLEGKHDKEIEDSLNQSDISRERWWPSLYKMVEGLAVMSRLARNRMQHPDYNL
EGKSFDWDNVEM

>AtABCC2

MCRKQDQNISTYLMFWWGGFWKIGNDCSQFVGPLLLNQLLKSMQEDAPAWMGYIYAFSIFVGVVFGVLCEAQYFQNVMRVGYRLRSALIAAVFRKSLRLTNEGRRKFQTGKI
TNLMTTDAESLQQICQSLHTMWSAPFRIIIALILLYQQLGVASLIGALLLVLMFPLQTVIISKMQKLTKEGLQRTDKRIGLMNEVLAAMDTVKCYAWENSFQSKVQTVRDDELSW
FRKSQLLGALNMFILNSIPVLVTIVSFGVFTLLGGDLTPARAFTSLSLFAVLRFPLFMLPNIITQVVNANVSLKRLEEVLATEERILLPNPPIEPGEPAISIRNGYFSWDSKGDRPTLSNI
NLDVPLGSLVAVVGSTGEGKTSLISAILGELPATSDAIVTLRGSVAYVPQVSWIFNATVRDNILFGSPFDREKYERAIDVTSLKHDLELLPGGDLTEIGERGVNISGGQKQRVSMAR
AVYSNSDVYIFDDPLSALDAHVGQQVFEKCIKRELGQKTRVLVTNQLHFLSQVDRIVLVHEGTVKEEGTYEELSSNGPLFQRLMENAGKVEEYSEENGEAEADQTAEQPVANGN
TNGLQMDGSDDKKSKEGNKKGGKSVLIKQEERETGVVSWRVLKRYQDALGGAWVVMMLLLCYVLTEVFRVTSSTWLSEWTDAGTPKSHGPLFYNLIYALLSFGQVLVTLTN
SYWLIMSSLYAAKKLHDNMLHSILRAPMSFFHTNPLGRIINRFAKDLGDIDRTVAVFVNMFMGQVSQLLSTVVLIGIVSTLSLWAIMPLLVLFYGAYLYYQNTAREVKRMDSISR
SPVYAQFGEALNGLSTIRAYKAYDRMADINGRSMDNNIRFTLVNMGANRWLGIRLETLGGLMIWLTASFAVMQNGRAENQQAFASTMGLLLSYALNITSLLTGVLRLASLAEN
SLNAVERVGNYIEIPPEAPPVIENNRPPPGWPSSGSIKFEDVVLRYRPQLPPVLHGVSFFIHPTDKVGIVGRTGAGKSSLLNALFRIVEVEKGRILIDDCDVGKFGLMDLRKVLGIIPQ
SPVLFSGTVRFNLDPFGEHNDADLWESLERAHLKDTIRRNPLGLDAEVSEAGENFSVGQRQLLSLSRALLRRSKILVLDEATAAVDVRTDALIQKTIREEFKSCTMLIIAHRLNTIID
CDKILVLDSGRVQEFSSPENLLSNEGSSFSKMVQSTGAANAEYLRSLVLDNKRAKDDSHHLQGQRKWLASSRWAAAAQFALAASLTSSHNDLQSLEIEDDSSILKRTNDAVVTL
RSVLEGKHDKEIAESLEEHNISREGWLSSLYRMVEGLAVMSRLARNRMQQPDYNFEGNTFDWDNVEM

>AtABCC3

MDFLGSTTGSGTLAMLFSFSESILPLDSRSFLLKPLFLRWLSGFLHSVLLLVLFFSWVRKKIRGDSGVTESLKDRRDFGFKSALFCSLALSLLNLVLMSLSGFYWYESGWLDNEQL
VSSLGFLLGMVSWGVLSICLHRCRDCEHKKAPFLLRLWLVFYLVVSCYSLVVDFVMYERRETVPVHLLVFDIVAFIAAVFLGYVAVLKKDRSNSNGVLEEPLLNGGDSRVGGD
DSVELNKTNGSGEATPYSRAGILSLLTFSWMSPLIDIGNKKTLDLEDVPQLHDTDSVVGLAPKFRSMLESPDGGERSGVTTFKLIKALYFTAQWEILVTAFFAFIYTVASYVGPALI
DTFVQYLNGRRQYNHEGYVLVITFFAAKIVECLSQRHWFFRLQKVGIRMRSALVAMIYEKGLTLSCQSKQGRTSGEIINFMTVDAERIGNFSWYMHDPWMVLLQVGLALWILY
RNLGLASIAALVATIIVMLINFPFGRMQERFQEKLMEAKDSRMKSTSEILRNMRILKLQGWEMKFLSKIFDLRKSEEGWLKKYVYNSAVISFVFWGAPTLVSVSTFGACILLGIPLE
SGKILSALATFRILQEPIYNLPDTISMIVQTKVSLDRLASYLCLDNLQPDIVERLPKGSSDVAVEVINSTLSWDVSSSNPTLKDINFKVFPGMKVAVCGTVGSGKSSLLSSLLGEVPK
VSGSLKVCGTKAYVAQSPWIQSGKIEDNILFGKPMERERYDKVLEACSLSKDLEILSFGDQTVIGERGINLSGGQKQRIQIARALYQDADIYLFDDPFSAVDAHTGSHLFKEVLLGL
LCSKSVIYVTHQVEFLPAADLILVMKDGRISQAGKYNDILNSGTDFMELIGAHQEALAVVDSVDANSVSEKSALGQENVIVKDAIAVDEKLESQDLKNDKLESVEPQRQIIQEEER
EKGSVALDVYWKYITLAYGGALVPFILLGQVLFQLLQIGSNYWMAWATPVSEDVQAPVKLSTLMIVYVALAFGSSLCILLRATLLVTAGYKTATELFHKMHHCIFRSPMSFFDS
TPSGRIMSRASTDQSAVDLELPYQFGSVAITVIQLIGIIGVMSQVSWLVFLVFIPVVAASIWYQRYYIAAARELSRLVGVCKAPLIQHFSETISGATTIRSFSQEFRFRSDNMRLSDGY
SRPKFYTAGAMEWLCFRLDMLSSLTFVFSLVFLVSIPTGVIDPSLAGLAVTYGLSLNTLQAWLIWTLCNLENKIISVERILQYASVPSEPPLVIESNRPEQSWPSRGEVEIRDLQVRY
APHMPLVLRGITCTFKGGLRTGIVGRTGSGKSTLIQTLFRIVEPSAGEIRIDGVNILTIGLHDLRLRLSIIPQDPTMFEGTMRSNLDPLEEYTDDQIWEALDKCQLGDEVRKKEQKLD
SSVSENGDNWSMGQRQLVCLGRVLLKRSKILVLDEATASVDTATDNLIQKTLREHFSDCTVITIAHRISSVIDSDMVLLLSNGIIEEYDTPVRLLEDKSSSFSKLVAEYTSRSSSSFD

>OsABCC9

MLGLAHLSVMYIGPSLVDRFVNFVRRGGELTEGLQLVVVLLAGKAAEALASHHYEFQGQKLGMRIHAALLAAVYRKSLRLSTGARRAHGAGAIVNYMEVDAEEVANVTHELH
NLWLMPLEIAVALTLLYTHLGPAVLTAVAAIAVVTVVVALANRRNLEYQFKFLGKRDERMKAITELLNYMRVIKLQGWEETFGGKIHELREAELGWLAKSMYFMCANTVVLW
SGPLAMTVLVFGTCVLTGVTLDAGKVFTATAFFHMLDGPMQSFPEAIASVTQATVSLGRLDRYLLDVELDDTTVERVDDAGINPDGVVVEVRDGVFAWDVRGKKENEEGDDN
EDDEEGEEEEEEKDVEETPVLETVLKGINIEVRRGELAAVVGTVGSGKSSLLSCIMGEMDKVSGKVRICGSTAYVAQTAWIQNGTIQENILFGQPMDAERYKEVLRSCSLEKDLE
MMEFGDQTEIGERGINLSGGQKQRIQLARAVYQNCDIYLLDDVFSAVDAHTGSSIFKECLRGMLKGKTILLVTHQVDFLHNVDNIFVMRDGMIVQSGKYDELLDAGSDFLALVA
AHDSSMELVDQSRQVVKTEYSQPKAVARIPSLRSRSIGKGEKVLVAPDIEAATSKIIREEERESGQVSWRVYKLYMTEAWGWWGVVGMLAFAIVWQVTEMASDYWLSYETSG
SIPFNPSLFIGVYVAIAAVSIILQVIKSLLETILGLQTAQIFFKKMFDSILHAPMSFFDTTPSGRILSRASSDQTTIDIVLSFFVGLTISMYISVLSTIIVTCQVAWPSVIAVIPLVLLNIWYR
NRYLATSRELTRLEGVTKAPVIDHFSETVLGATTIRCFKKDKEFFQENLDRINSSLRMYFHNYAANEWLGFRLELIGTLVLAITAFLMISLPSNFIKKEFVGMSLSYGLSLNSLVYFA
ISISCMLENDMVAVERVNQFSTLPSEAVWKIEDHLPSPNWPTHGDIDIDDLKVRYRPNTPLILKGITVSISGGEKIGVVGRTGSGKSTLIQALFRLVEPVQGTMIIDGIDICTLGLHDL
RSRFGIIPQEPVLFEGTIRSNIDPIGQYSDAEIWRALEGCQLKDVVASKPQKLDALVADSGENWSVGQRQLLCLGRVILKRTRILFMDEATASVDSQTDATIQKITRQEFSSCTIISIA
HRIPTVMDCDRVLVLDAGLVKEFDSPSRLIEQPSLFGAMVEEYANRSSNL

>OsMTP1

MDSHNSAPPQIAEVRMDISSSTSVAAGNKVCRGAACDFSDSSNSSKDARERMASLRKLIIAVILCIIFMAVEVVGGIKANSLAILTDAAHLLSDVAAFAISLFSLWAAGWEATPQQS
YGFFRIEILGALVSIQLIWLLAGILVYEAIVRLINESGEVQGSLMFAVSAFGLFVNIIMAVLLGHDHGHGHGHGHGHGHSHDHDHGGSDHDHHHHEDQEHGHVHHHEDGHGNSIT
VNLHHHPGTGHHHHDAEEPLLKSDAGCDSTQSGAKDAKKARRNISIHSAYLHVLGDSIQSIGVMIGGAIIWYKPEWKIIDLICTLIFSVIVLFTTIKMLRNILEVLMESTPREIDATSL
ENGLRDMDGVVAVHELHIWAITVGKVLLACHVTITQDADADQMLDKVIGYIKSEYNISHVTIQIERE

>AtOPT4

MATADEFSDEDTSPIEEVRLTVTNTDDPTLPVWTFRMWFLGLISCSLLSFLNQFFSYRTEPLVITQITVQVATLPIGHFLAKVLPKTRFGLPGCGSARFSLNPGPFNMKEHVLISIFAN
AGSAFGSGSAYAVGIITIIKAFYGRSISFIAGWLLIITTQVLGYGWAGLLRKYVVEPAHMWWPSTLVQVSLFRALHEKDDQRMTRAKFFVIALVCSFGWYIVPGYLFTTLTSISWV
CWAFPRSVTAQQIGSGMRGLGLGAFTLDWTAVASFLFSPLISPFFAIANVFIGYVLLIYFVLPLAYWGFDSYNATRFPIFSSHLFTSVGNTYDIPAIVNDNFELDLAKYEQQGRINLS
MFFALTYGLGFATIASTLTHVALFYGKEISERFRVSYKGKEDIHTRLMKRYKDIPSWWFYSMLAATLLISLALCVFLNDEVQMPWWGLVFASAMAFVFTLPISIITATTNQTPGLN
IITEYAMGLIYPGRPIANVCFKVYGYMSMAQAVSFLNDFKLGHYMKIPPRSMFLVQFIGTILAGTINITVAWWQLNSIKNICQEELLPPNSPWTCPGDRVFFDASVIWGLVGPKRIF
GSQGNYAAMNWFFLGGALGPVIVWSLHKAFPKRSWIPLVNLPVLLGATAMMPPATAVNYNSWILVGTIFNLFVFRYRKSWWQRYNYVLSAAMDAGVAFMAVLLYFSVGMEE
KSLDWWGTRGEHCDLAKCPTARGVIVDGCPVK

>AtOPT6

MGEIATEFTPVMDDDDDRCVVPEVELTVPKTDDSTLPVLTFRMWVLGIGACIVLSFINQFFWYRTMPLSITGISAQIAVVPLGHLMARVLPTKRFLEGTRFQFTLNPGAFNVKEHV
LITIFANSGAGSVYATHILSAIKLYYKRSLPFLPAFLVMITTQILGFGWAGLFRKHLVEPGEMWWPSNLVQVSLFGALHEKEKKSRGGMSRTQFFLIVLVASFAYYIFPGYLFTMLT
SISWVCWLNPKSILVNQLGSGEHGLGIGSIGFDWVTISAYLGSPLASPLFASVNVAIGFVLVMYIVTPVCYWLNIYDAKTFPIFSSQLFMGNGSRYDVLSIIDSKFHLDRVVYSRTGS
INMSTFFAVTYGLGFATLSATIVHVLVFNGSDLWKQTRGAFQKNKKMDIHTRIMKKNYREVPLWWFLVILLLNIALIMFISVHYNATVQLPWWGVLLACAIAISFTPLIGVIAATT
NQAPGLNIITEYVIGYIYPERPVANMCFKVYGYISMTQALTFISDFKLGHYMKIPPRSMFMAQVAGTLVAVVVYTGTAWWLMEEIPHLCDTSLLPSDSQWTCPMDRVFFDASVI
WGLVGPRRVFGDLGEYSNVNWFFLVGAIAPLLVWLATKMFPAQTWIAKIHIPVLVGATAMMPPATAVNFTSWLIVAFIFGHFIFKYRRVWWTKYNYVLSGGLDAGSAFMTILL
FLALGRKGIEVQWWGNSGDRDTCPLASCPTAKGVVVKGCPVF

>AtHMA2

MASKKMTKSYFDVLGICCTSEVPLIENILNSMDGVKEFSVIVPSRTVIVVHDTLILSQFQIVKALNQAQLEANVRVTGETNFKNKWPSPFAVVSGILLLLSFFKYLYSPFRWLAVA
AVVAGIYPILAKAVASLARFRIDINILVVVTVGATIGMQDYTEAAVVVFLFTIAEWLQSRASYKASAVMQSLMSLAPQKAVIAETGEEVEVDELKTNTVIAVKAGETIPIDGVVV
DGNCEVDEKTLTGEAFPVPKLKDSTVWAGTINLNGYITVNTTALAEDCVVAKMAKLVEEAQNSKTETQRFIDKCSKYYTPAIILISICFVAIPFALKVHNLKHWVHLALVVLVSA
CPCGLILSTPVATFCALTKAATSGLLIKGADYLETLAKIKIVAFDKTGTITRGEFIVMDFQSLSEDISLQSLLYWVSSTESKSSHPMAAAVVDYARSVSVEPKPEAVEDYQNFPGEGI
YGKIDGKEVYIGNKRIASRAGCLSVPDIDVDTKGGKTIGYVYVGETLAGVFNLSDACRSGVAQAMKELKSLGIKIAMLTGDNHAAAMHAQEQLGNAMDIVRAELLPEDKSEIIK
QLKREEGPTAMVGDGLNDAPALATADIGISMGVSGSALATETGNIILMSNDIRRIPQAIKLAKRAKRKVVENVVISITMKGAILALAFAGHPLIWAAVLADVGTCLLVILNSMLLL
SDKHKTGNKCYRESSSSSVLIAEKLEGDAAGDMEAGLLPKISDKHCKPGCCGTKTQEKAMKPAKASSDHSHSGCCETKQKDNVTVVKKSCCAEPVDLGHGHDSGCCGDKSQQ
PHQHEVQVQQSCHNKPSGLDSGCCGGKSQQPHQHELQQSCHDKPSGLDIGTGPKHEGSSTLVNLEGDAKEELKVLVNGFCSSPADLAITSLKVKSDSHCKSNCSSRERCHHGSN
CCRSYAKESCSHDHHHTRAHGVGTLKEIVIE

>AtHMA4

MALQNKEEEKKKVKKLQKSYFDVLGICCTSEVPIIENILKSLDGVKEYSVIVPSRTVIVVHDSLLISPFQIAKALNEARLEANVRVNGETSFKNKWPSPFAVVSGLLLLLSFLKFVY
SPLRWLAVAAVAAGIYPILAKAFASIKRPRIDINILVIITVIATLAMQDFMEAAAVVFLFTISDWLETRASYKATSVMQSLMSLAPQKAIIAETGEEVEVDEVKVDTVVAVKAGETI
PIDGIVVDGNCEVDEKTLTGEAFPVPKQRDSTVWAGTINLNGYICVKTTSLAGDCVVAKMAKLVEEAQSSKTKSQRLIDKCSQYYTPAIILVSACVAIVPVIMKVHNLKHWFHLA
LVVLVSGCPCGLILSTPVATFCALTKAATSGLLIKSADYLDTLSKIKIVAFDKTGTITRGEFIVIDFKSLSRDINLRSLLYWVSSVESKSSHPMAATIVDYAKSVSVEPRPEEVEDYQN
FPGEGIYGKIDGNDIFIGNKKIASRAGCSTVPEIEVDTKGGKTVGYVYVGERLAGFFNLSDACRSGVSQAMAELKSLGIKTAMLTGDNQAAAMHAQEQLGNVLDVVHGDLLPED
KSRIIQEFKKEGPTAMVGDGVNDAPALATADIGISMGISGSALATQTGNIILMSNDIRRIPQAVKLARRARRKVVENVCLSIILKAGILALAFAGHPLIWAAVLVDVGTCLLVIFNS
MLLLREKKKIGNKKCYRASTSKLNGRKLEGDDDYVVDLEAGLLTKSGNGQCKSSCCGDKKNQENVVMMKPSSKTSSDHSHPGCCGDKKEEKVKPLVKDGCCSEKTRKSEGD
MVSLSSCKKSSHVKHDLKMKGGSGCCASKNEKGKEVVAKSCCEKPKQQVESVGDCKSGHCEKKKQAEDIVVPVQIIGHALTHVEIELQTKETCKTSCCDSKEKVKETGLLLSSE
NTPYLEKGVLIKDEGNCKSGSENMGTVKQSCHEKGCSDEKQTGEITLASEEETDDQDCSSGCCVNEGTVKQSFDEKKHSVLVEKEGLDMETGFCCDAKLVCCGNTEGEVKEQC
RLEIKKEEHCKSGCCGEEIQTGEITLVSEEETESTNCSTGCCVDKEEVTQTCHEKPASLVVSGLEVKKDEHCESSHRAVKVETCCKVKIPEACASKCRDRAKRHSGKSCCRSYAK
ELCSHRHHHHHHHHHHHVSA

>OsHMA2

MAAEGGRCQKSYFDVLGICCPSEVPLVEKLLQPLEGVQKVTVIVPSRTVIVVHDVDAISQSQIVKALNQARLEASVRAYGNGSEKITNKWPSPYVLLCGLLLVVSLFEHFWHPLK
WFALVAAAAGLPPIVLRSIAAIRRLTLDVNILMLIAVAGAIALKDYSEAGFIVFLFTTAEWLETRASHKATAGMSALMSMAPQKAILAETGEVVAARDVKVNTVIAVKAGEVIPID
GVVVDGRSEVDESTLTGESFPVSKQPDSQVWAGTLNIDGYIAVRTTAMADNSAVAKMARLVEEAQNSRSSTQRLIDTCAKYYTPAVVVMAGSVAAIPAIAKAHNLKHWFQLA
LVLLVSACPCALVLSTPIATFCALLRAARTGLLIKGGDVLESLASIKVAAFDKTGTITRGEFSVEEFQPVGERVSLQQLLYWVSSVESRSSHPMASVLVDYAQSKSVEPKSENVSEF
QIYPGEGIYGEIDGAGIYIGNKRILSRASCETVPDMKDMKGVTIGYVACNNELIGVFTLSDACRTGSAEAIKELRSLGIKSVMLTGDSSAAATYAQNQLGNILAEVHAELLPEDKVR
IVGELKEKDGPTLMVGDGMNDAPALAKADVGVSMGVSGSAVAMETSHVALMSNDIRRIPKAVRLARRTHRTIIVNIIFSVITKLAIVGLAFAGHPLIWAAVLADVGTCLLVIMYS
MLLLREKDSRKAKKCAASHHGSPKKCCSSSHHGSHAKKNHGVSHHCSDGPCKSMVSCKESSVAKNACHDHHHEHNHHEEPAHKHSSNQHGCHDHSHGHSNCKEPSNQLITNK
HACHDGHNHCADTSNLHDTKKHDCHGHEHSTCKEELNALPPTNDHACHGHEHSHCEEPVALHSTGEHACHEHEHEHIHCDEPIGSHCADKHACHDHEQVHEHHCCDEQQTPH
TADLHPCHDHDHDNLEVEEVKDCHAEPPHHHNHCCHEPHDQVKNDTHPVQEHSISIEESSDHHEHHHNEEHKAEDCGHHPKPKDCAPPPTDCISRNCCSNTSKGKDICSSLHRD
HHTSQASRCCRSYVKCSRPSRSCCSHSIVKLPEIVVE

>AtPCR1

MEAQLHAKPHAQGEWSTGFCDCFSDCRNCCITLCCPCITFGQVAEIVDRGSKSCCAAGALYMLIDLITSCGRMYACFYSGKMRAQYNIKGDGGCTDCLKHFCCNLCALTQQYRE
LKHRGFDMSLGWAGNAEKQQNQGGVAMGAPAFQGGMTR

>OsPCR1

MYPPDPSKSGESSAAAPEPVTGIPVGMFYPAPPMERVVSCRMAPAAGGAWTTALCDCADDCNTCCMACWCPCIPVGQIAEIVDRGSSSCALNAVLYCLVFHVSAGMCQWVYS
CAYRARLRAAYDLPETPCSDCLVTFCCQTCSIAQMHRELKNRGHDPNLG

>OsPCR3

MYPSAPPDAYNKYSAGAPPTAPPPATYQLPTMNTPRTGGGLTRWSTGLFHCMDDPGNCLITCVCPCITFGQVADIVDKGTCPCLASGTAYALLCASGMGCLYSCFYRSKMRAQF
DLDEGDCPDFLVHFCCEYCALCQEYRELKNRGFDLGIGWAANVDRQRRGVTGASVMGAPGVPVGMMR

>AtABCG36

MDYNPNLPPLGGGGVSMRRSISRSVSRASRNIEDIFSSGSRRTQSVNDDEEALKWAAIEKLPTYSRLRTTLMNAVVEDDVYGNQLMSKEVDVTKLDGEDRQKFIDMVFKVAEQD
NERILTKLRNRIDRVGIKLPTVEVRYEHLTIKADCYTGNRSLPTLLNVVRNMGESALGMIGIQFAKKAQLTILKDISGVIKPGRMTLLLGPPSSGKTTLLLALAGKLDKSLQVSGDI
TYNGYQLDEFVPRKTSAYISQNDLHVGIMTVKETLDFSARCQGVGTRYDLLNELARREKDAGIFPEADVDLFMKASAAQGVKNSLVTDYTLKILGLDICKDTIVGDDMMRGISG
GQKKRVTTGEMIVGPTKTLFMDEISTGLDSSTTFQIVKCLQQIVHLNEATVLMSLLQPAPETFDLFDDIILVSEGQIVYQGPRDNILEFFESFGFKCPERKGTADFLQEVTSKKDQEQ
YWVNPNRPYHYIPVSEFASRYKSFHVGTKMSNELAVPFDKSRGHKAALVFDKYSVSKRELLKSCWDKEWLLMQRNAFFYVFKTVQIVIIAAITSTLFLRTEMNTRNEGDANLYI
GALLFGMIINMFNGFAEMAMMVSRLPVFYKQRDLLFYPSWTFSLPTFLLGIPSSILESTAWMVVTYYSIGFAPDASRFFKQFLLVFLIQQMAASLFRLIASVCRTMMIANTGGALT
LLLVFLLGGFLLPKGKIPDWWGWAYWVSPLTYAFNGLVVNEMFAPRWMNKMASSNSTIKLGTMVLNTWDVYHQKNWYWISVGALLCFTALFNILFTLALTYLNPLGKKAGL
LPEEENEDADQGKDPMRRSLSTADGNRRGEVAMGRMSRDSAAEASGGAGNKKGMVLPFTPLAMSFDDVKYFVDMPGEMRDQGVTETRLQLLKGVTGAFRPGVLTALMGVS
GAGKTTLMDVLAGRKTGGYIEGDVRISGFPKVQETFARISGYCEQTDIHSPQVTVRESLIFSAFLRLPKEVGKDEKMMFVDQVMELVELDSLRDSIVGLPGVTGLSTEQRKRLTIA
VELVANPSIIFMDEPTSGLDARAAAIVMRAVRNTVDTGRTVVCTIHQPSIDIFEAFDELMLMKRGGQVIYAGPLGQNSHKVVEYFESFPGVSKIPEKYNPATWMLEASSLAAELKL
SVDFAELYNQSALHQRNKALVKELSVPPAGASDLYFATQFSQNTWGQFKSCLWKQWWTYWRSPDYNLVRFIFTLATSLLIGTVFWQIGGNRSNAGDLTMVIGALYAAIIFVGIN
NCSTVQPMVAVERTVFYRERAAGMYSAMPYAISQVTCELPYVLIQTVYYSLIVYAMVGFEWKAEKFFWFVFVSYFSFLYWTYYGMMTVSLTPNQQVASIFASAFYGIFNLFSGF
FIPRPKIPKWWIWYYWICPVAWTVYGLIVSQYGDVETRIQVLGGAPDLTVKQYIEDHYGFQSDFMGPVAAVLIAFTVFFAFIFAFCIRTLNFQTR

>OsABCG36

MDAAGEIQKVASMRLGGSMRGDSGSMWRRGDDVFSRSSREEDDEEALRWAALEKLPTYDRVRRAILPLGGDDGAGDGGGKGVVDVHGLGPRERRALLERLVRVADEDNEKF
LLKLKDRVDRVGIDMPTIEVRFEHLEAEVRVGNSGLPTVLNSITNTLEEAGNALGILPNRKQTMPVLHDVSGIIKPRRMTLLLGPPGSGKTTLLLALAGRLGKDLKASGKVTYNG
HGMEEFVPERTAAYISQHDLHIGEMTVRETLAFSARCQGVGSRFDMLTELSRREKAANIKPDADIDAFMKAAAMGGQEANVNTDYILKILGLEICADTMVGDEMLRGISGGQRK
RVTTGEMLVGPARALFMDEISTGLDSSTTFQIVNSLRQTVHILGGTAVISLLQPAPETYNLFDDIILLSDGQIVYQGPREDVLEFFESMGFKCPDRKGVADFLQEVTSKKDQRQYW
ARHDKPYRFVTVKEFVSAFQSFHTGRAIANELAVPFDKSKSHPAALATTRYGAPGKELLKANIDREILLMKRNSFVYMFRTFQLMVVSLIAMTLFFRTKMKRDSVTSGGIYMGA
LFFGVLMIMFNGFSELALTVFKLPVFFKQRDLLFYPAWSYTIPSWILKIPITFIEVGGYVFLTYYVIGFDSNVGSFFKQYLLMLAINQMAGSLFRFIGGAARNMMIVANVFASFMLLI
FMVLGGFILAREQVKKWWIWGYWISPMMYAQNAISVNELMGHSWNKIVNSSASNETLGVQVLKSRGVFPEARWYWIGFGAMIGFTILFNALFTLALTYLRPYGNSRQSVSEEK
RANLNGEIVGDVHLSSGSTRRPMGGNGTENDSTIVDDDTEVTQRGMVLPFTPLSLSFDNVRYSVDMPQEMKAQGVADDRLELLKGVSGSFRPGVLTALMGVSGAGKTTLMDV
LAGRKTGGYIEGSINISGYPKKQETFARVSGYCEQNDIHSPQVTVYESLLFSAWLRLPEDVDSNTRKMFIEEVMELVELKSLRDALVGLPGVNGLSTEQRKRLTIAVELVANPSIIF
MDEPTSGLDARAAAIVMRTVRNTVNTGRTVVCTIHQPSIDIFEAFDELFLMKRGGEEIYAGPLGHHSSELIKYFESIPGVSKIKDGYNPATWMLEVTTIGQEQALGVDFSDIYKKSE
LYQRNKALIKDLSQPAPDSSDLYFPTQYSQSLTQCMACLWKQNLSYWRNPPYNAVRFFFFTTVIALLFGTIFWLGGKVTKSQDLFNAMGSMYAAVLFIGVMNCTSVQPVVAVE
RTVFYRERAAGMYSAFPYAFGQVVIEIPYTLVQATVYGIIVYAMIGFEWTAAKFFWYLFFMVFTLLYFTFYGMMAVGLTPNYHIASIVSSAFYAIWNLFSGFVIPRPRVPIWWRW
YCWACPVAWTLYGLVVSQFGDIETPMEDGTPVKVFVENYFGFKHSWLGWVATVVAAFAFLFASLFGFAIMKFNFQKR

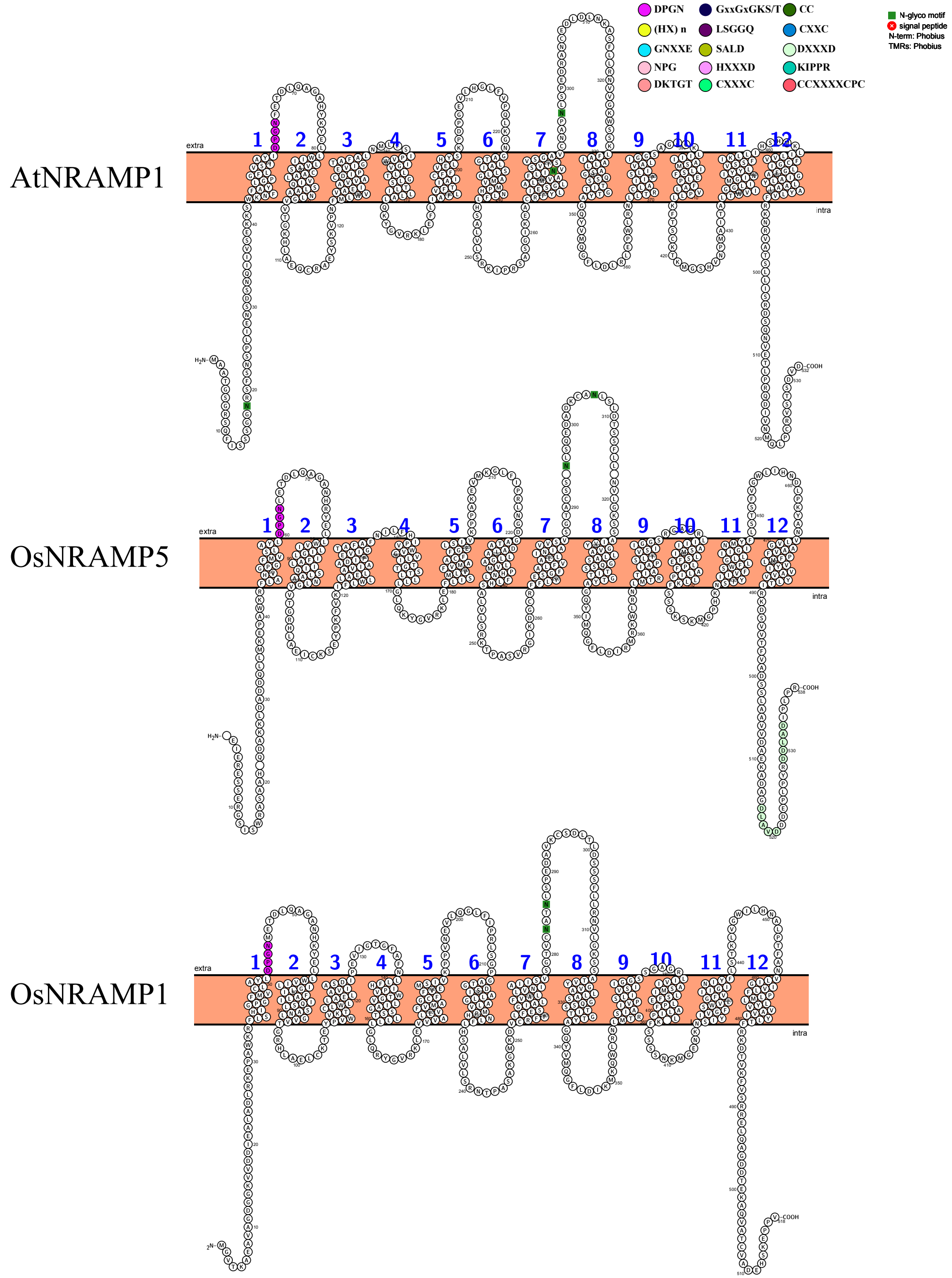

Figure S1: Specific motifs and transmembrane structural features of NRAMP proteins involved in Cd absorption, Different residues are indicated by different colors. The DPGN is near to TMD1.

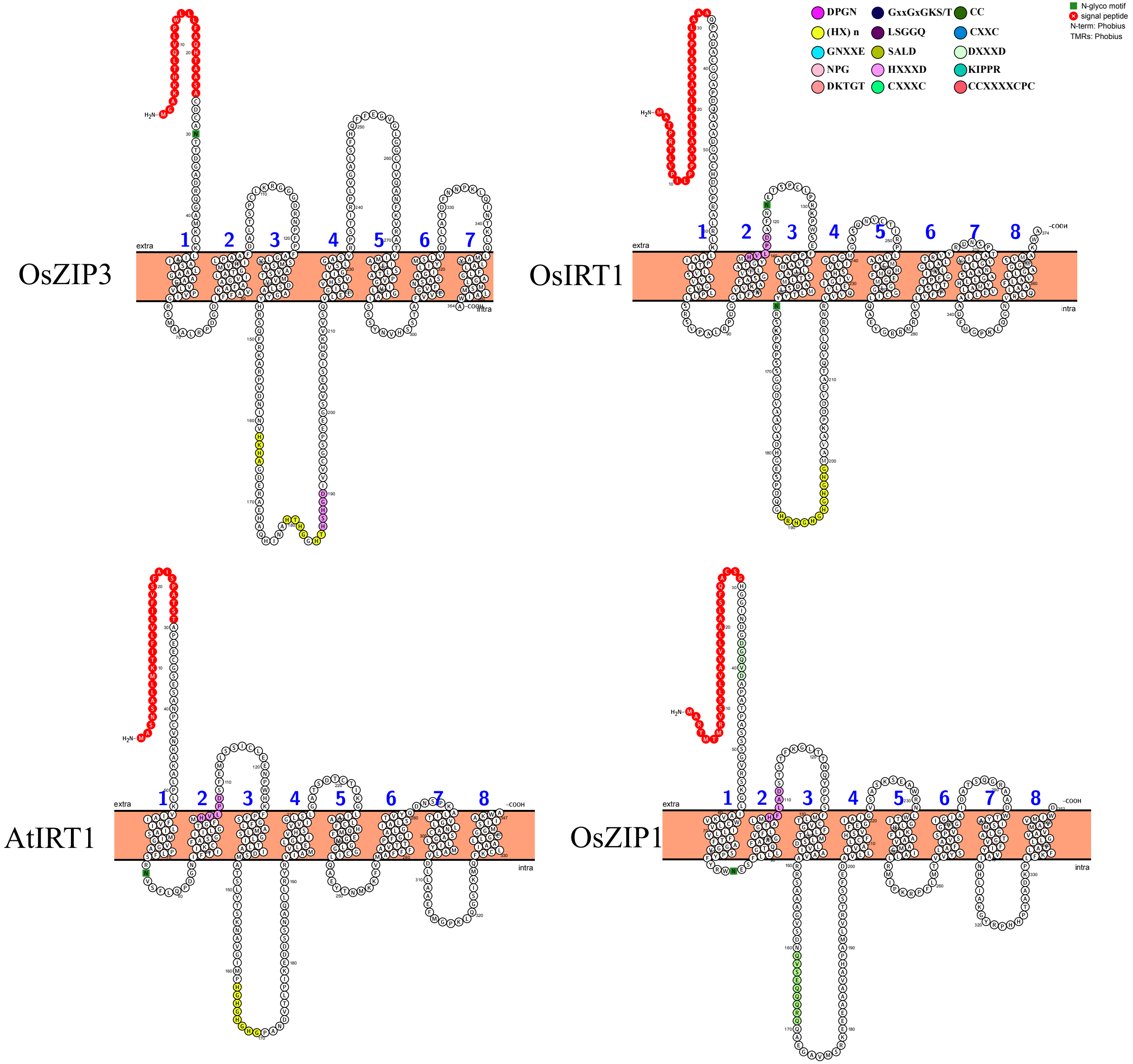

Figure S2: Specific motifs and transmembrane structural features of ZIP proteins involved in Cd absorption. Different residues are indicated by different colors. 8 transmembrane domains, with signal peptide and DXXXD residues at the N-terminus; The (HX)n residues between TMD3-TMD4.

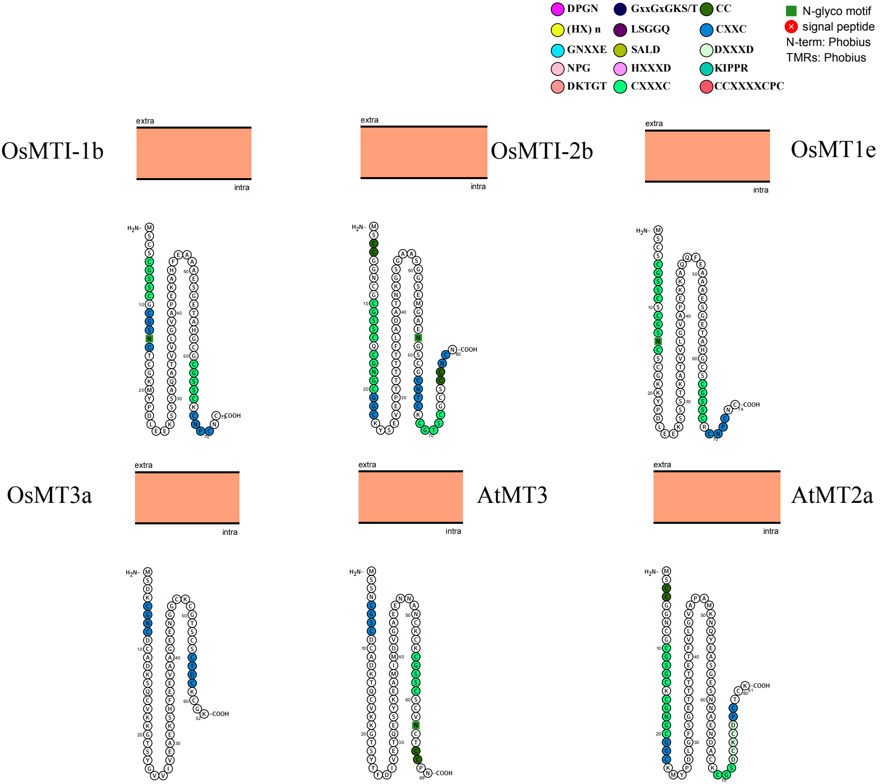

Figure S3: Specific motifs and transmembrane structural features of MT protein involved in Cd chelation. Different residues are indicated by different colors. They have no transmembrane domain, but contain cysteine residues.

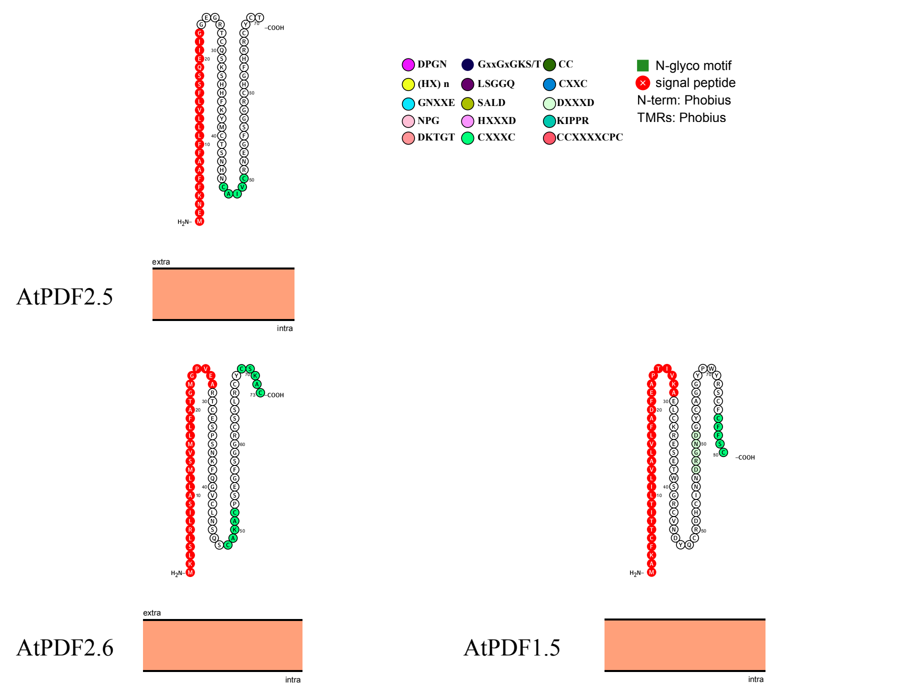

Figure S4: Specific motifs and transmembrane structural features of PDF protein involved in Cd chelation. Different residues are indicated by different colors. They have no transmembrane domain, but contain cysteine residues.

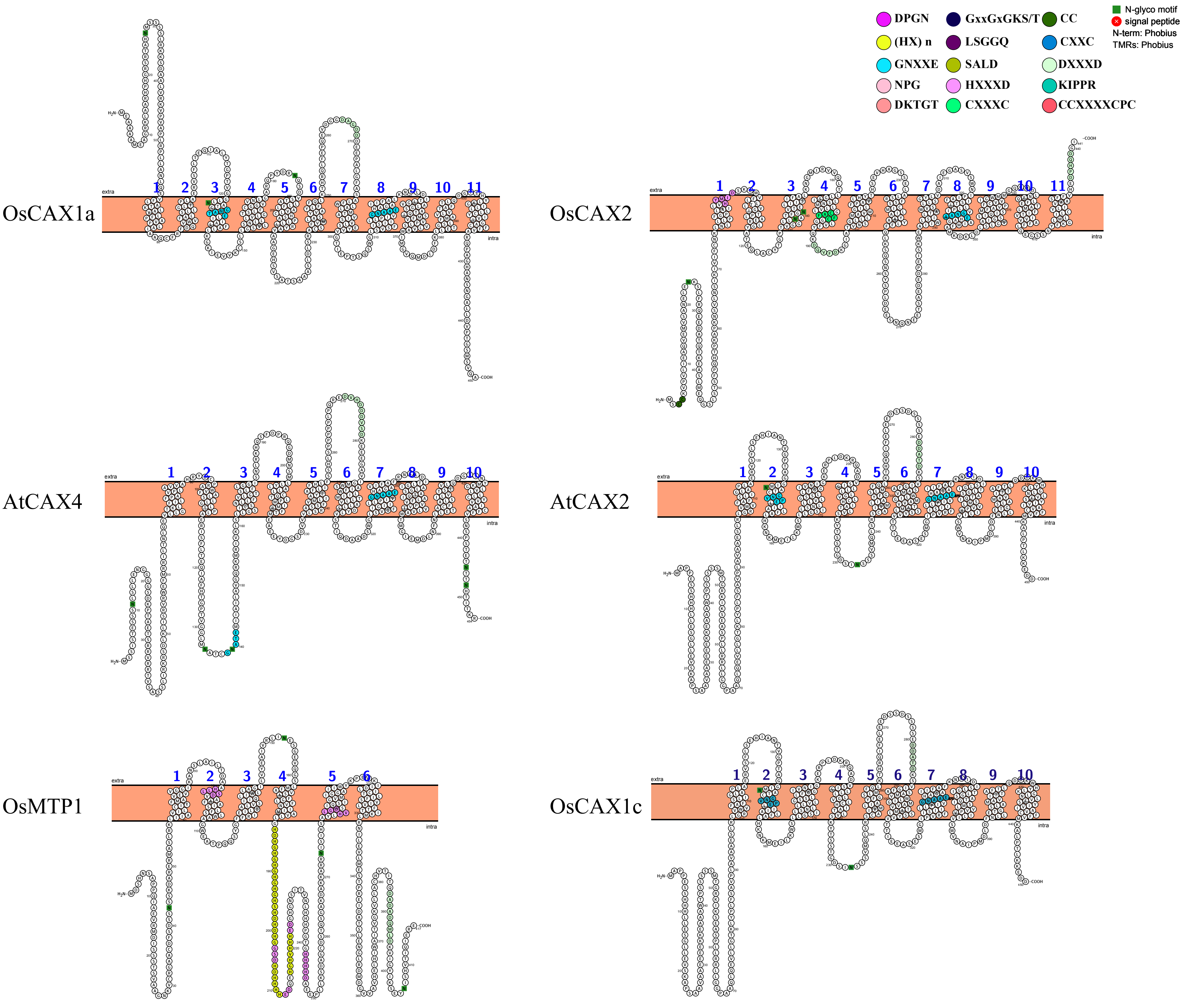

Figure S5: The specific motifs and transmembrane structural features of the vacuolar protein CAX, MTP family members. Different residues are indicated by different colors. CAX family members contain GNXXE residues, and H/DXXXD residues are widely distributed in these two family members.

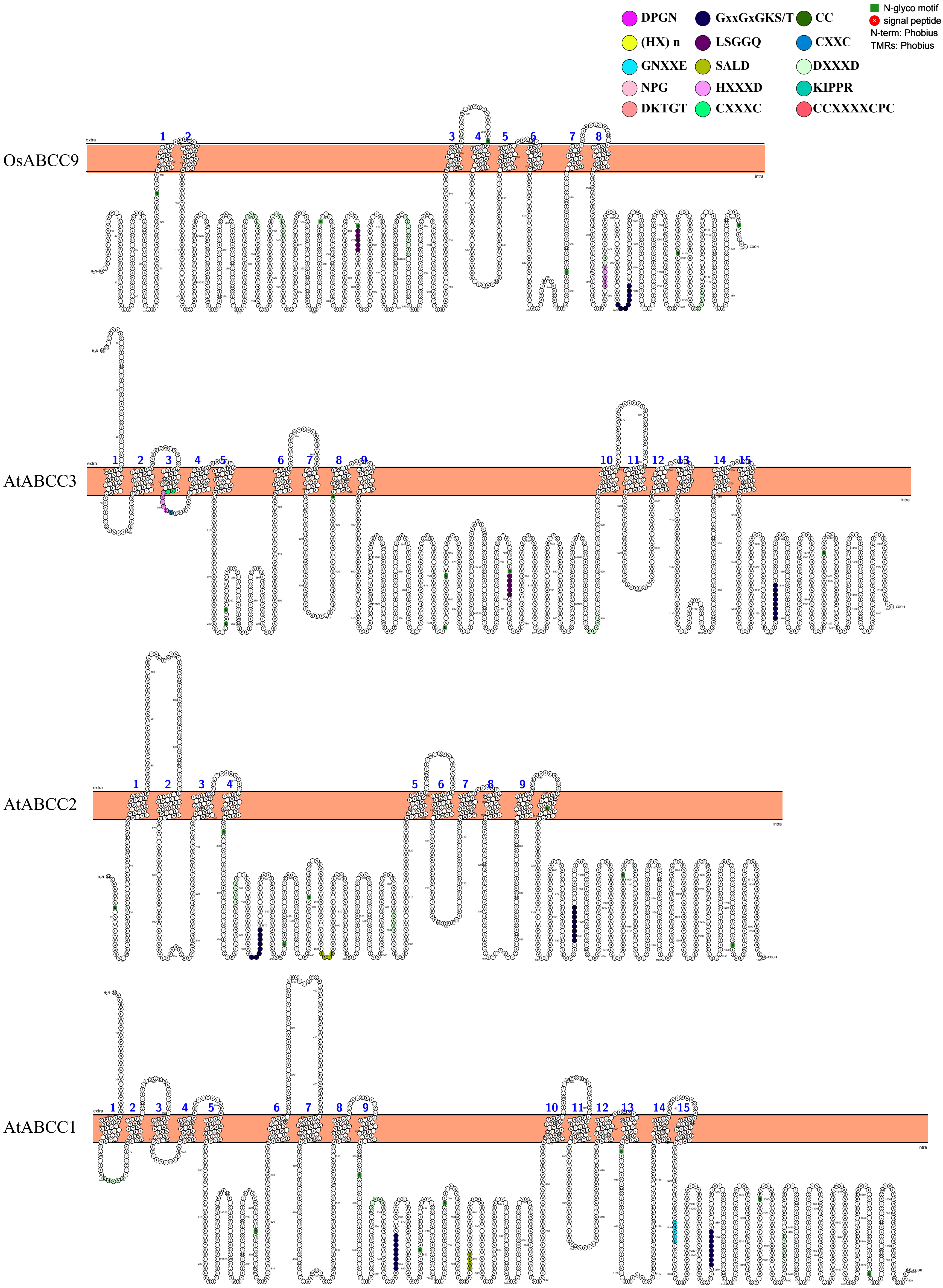

Figure S6: The specific motifs and transmembrane structural features of the vacuolar protein ABCC family members. Different residues are indicated by different colors. With cysteine-rich loop, Q-loop, Walker B motif, H/DXXXD residues.

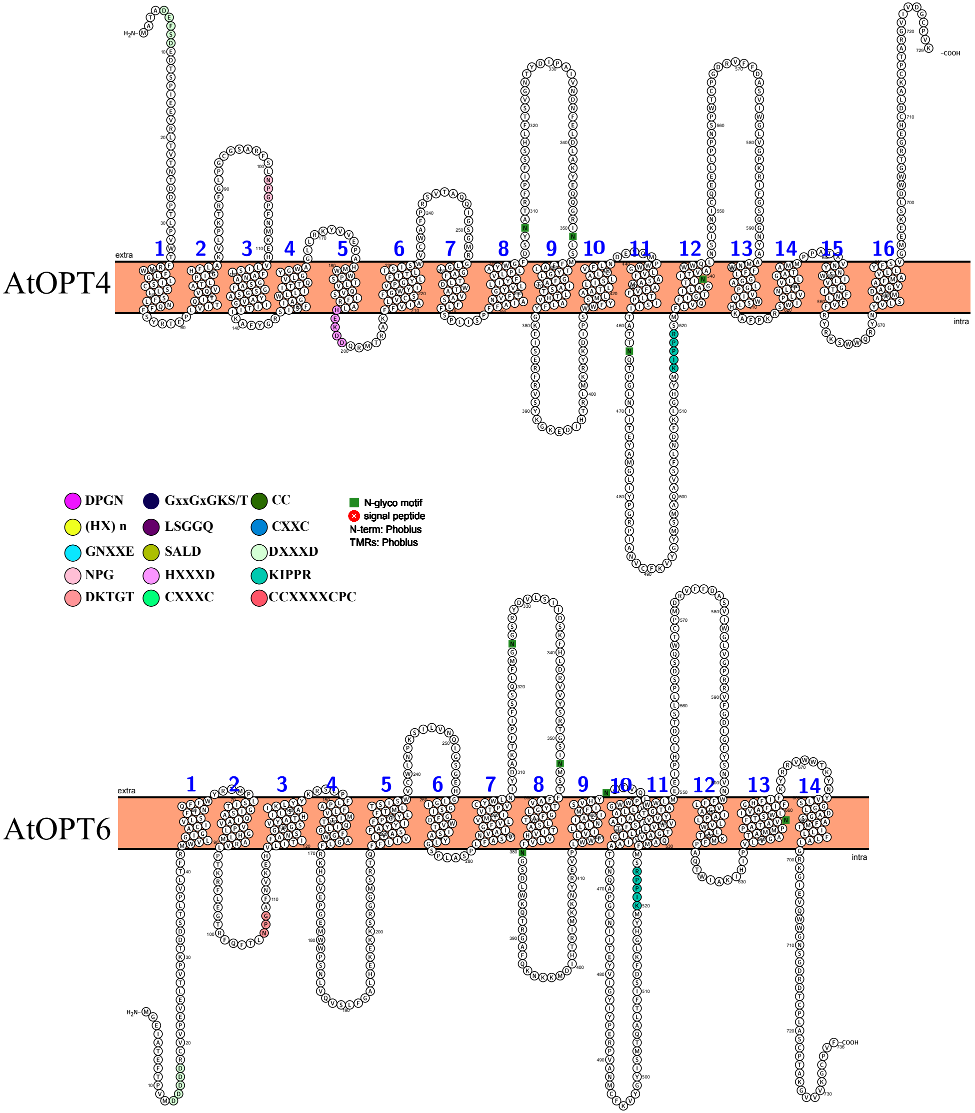

Figure S7: Specific motifs and transmembrane structural features of members of the OPT family of long-distance transport proteins.Different residues are indicated by different colors. They contain KIPPR residues unique to this family.

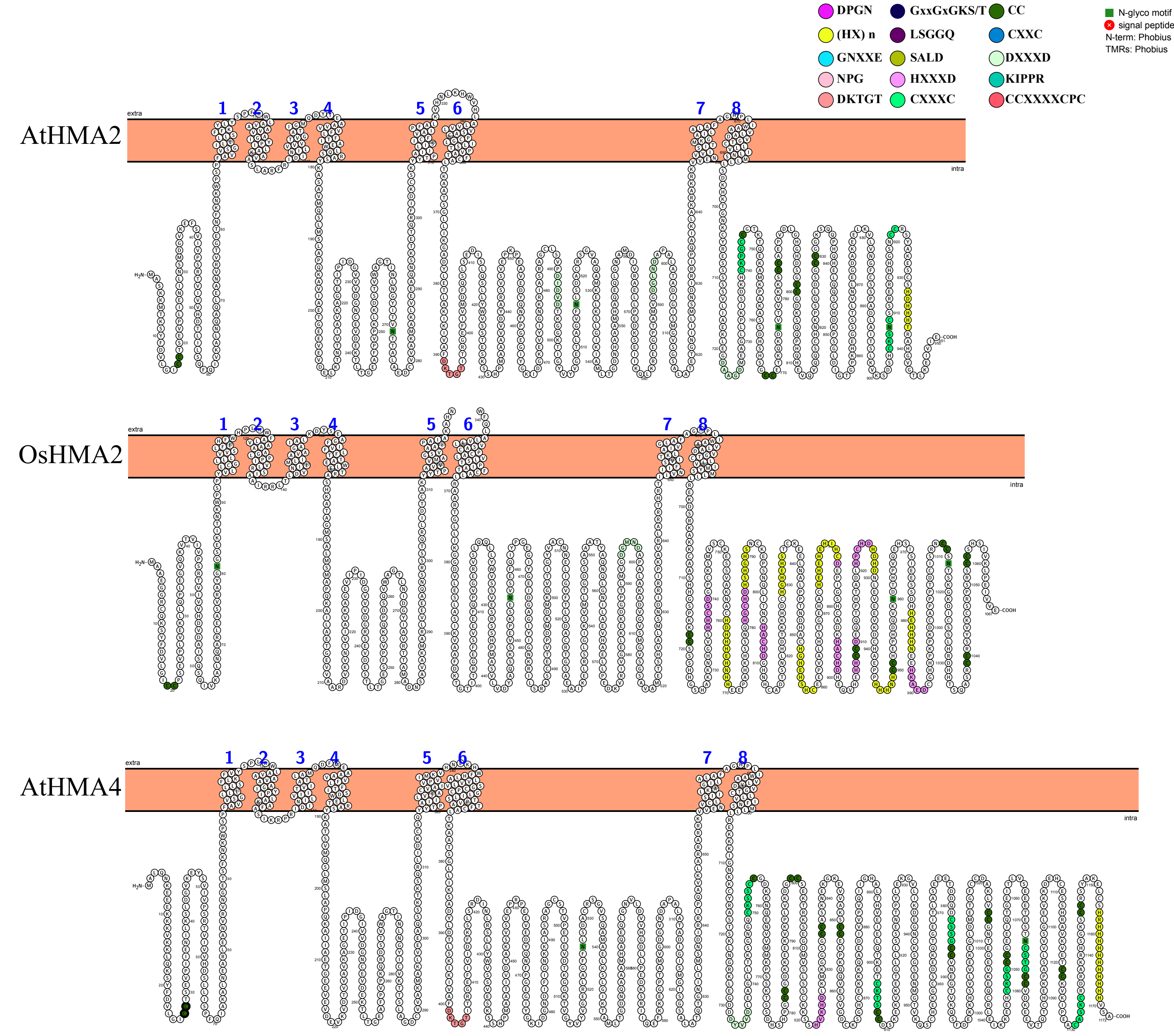

Figure S8: Specific motifs and transmembrane structural features of members of the HMA family of long-distance transport proteins. Different residues are indicated by different colors. They have (HX)n and cysteine related residues.

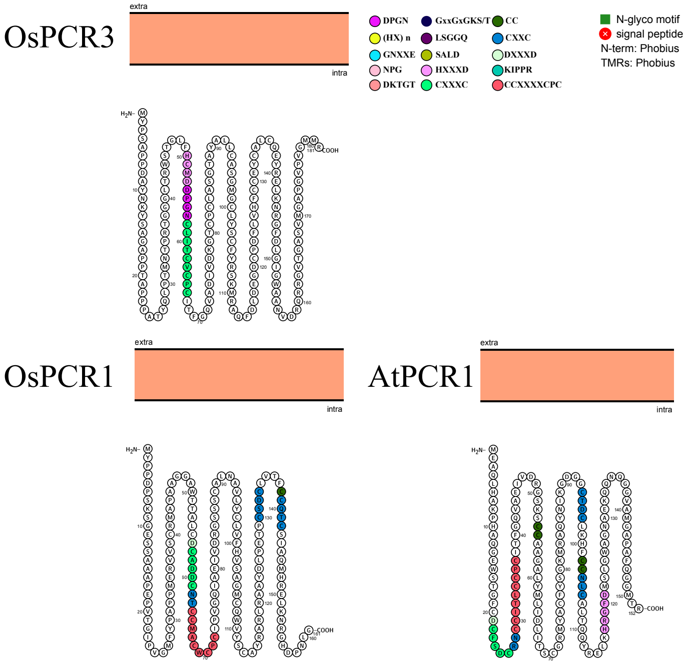

Figure S9: Specific motifs and transmembrane structural features of members of the PCR family of efflux proteins. Different residues are indicated by different colors. They have CCXXXXCPC and cysteine related residues.

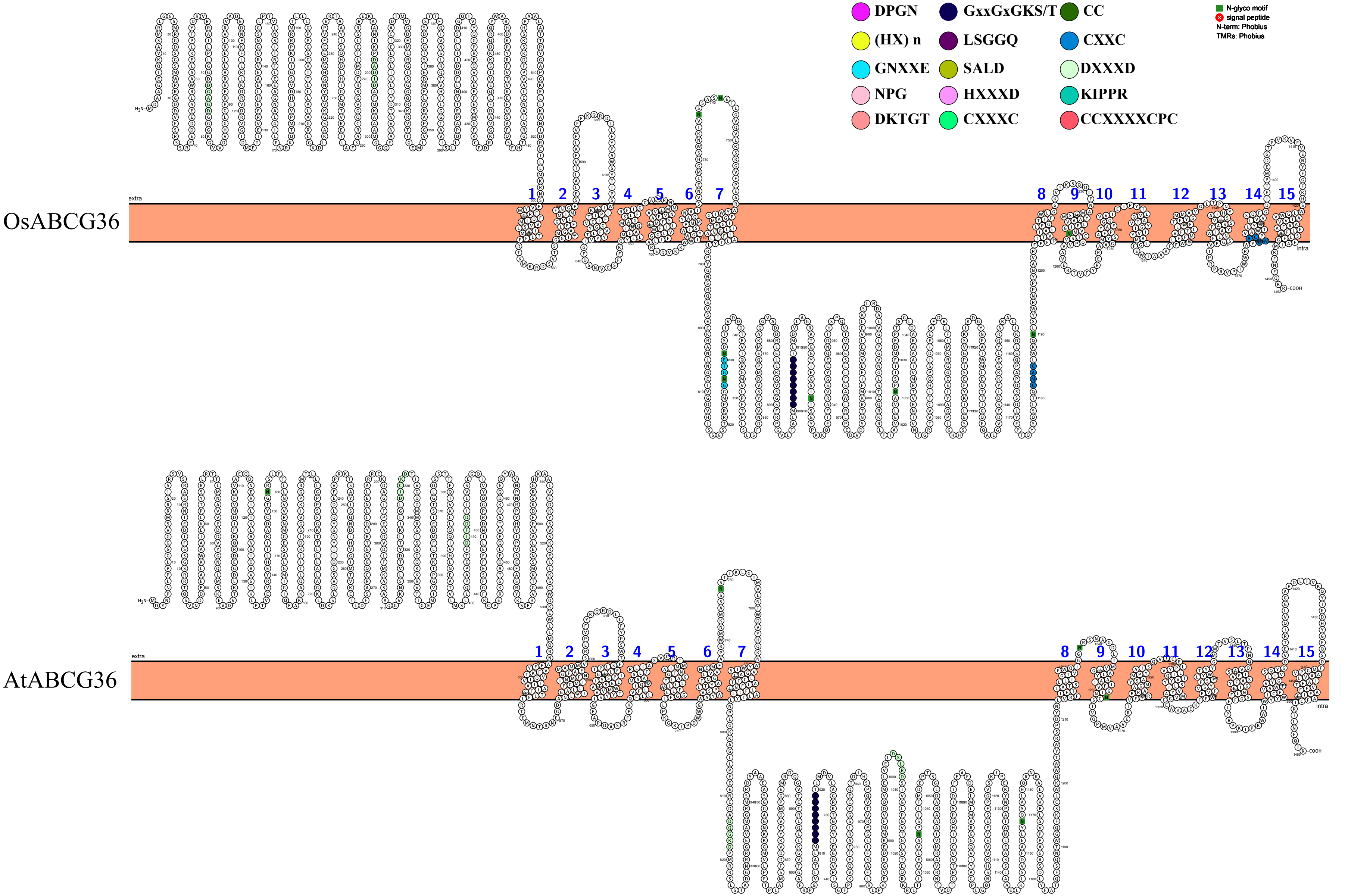

Figure S10: Specific motifs and transmembrane structural features of members of the ABCG family of efflux proteins. Different residues are indicated by different colors. They have 15 transmembrane domains and GxxGxGKS/T residues.
